# The social networks of hospital staff: A realist synthesis

**DOI:** 10.1177/13558196221076699

**Published:** 2022-05-05

**Authors:** Claire Blacklock, Amy Darwin, Mike English, Jacob McKnight, Lisa Hinton, Elinor Harriss, Geoff Wong

**Affiliations:** 1 Nuffield Department of Medicine, 6396 University of Oxford, UK; 2 8955 Oxford Health NHS Foundation Trust, UK; 3 The Healthcare Improvement Studies Institute, 2152 University of Cambridge, UK; 4 Bodleian Health Care Libraries, 6396 University of Oxford, UK; 5 Nuffield Department of Primary Care Health Sciences, 6396 University of Oxford, UK

## Abstract

**Objectives:**

The social ties people have with one another are known to influence behaviour, and how information is accessed and interpreted. It is unclear, however, how the social networks that exist in multi-professional health care workplaces might be used to improve quality in hospitals. This paper develops explanatory theory using realist synthesis to illuminate the details and significance of the social ties between health care workers. Specifically we ask: How, why, for whom, to what extent and in what context, do the social ties of staff within a hospital influence quality of service delivery, including quality improvement?

**Methods:**

From a total of 75 included documents identified through an extensive systematic literature search, data were extracted and analysed to identify emergent explanatory statements.

**Results:**

The synthesis found that within the hospital workforce, an individual’s place in the social whole can be understood across four identified domains: (1) social group, (2) hierarchy, (3) bridging distance and (4) discourse. Thirty-five context-mechanism-outcome configurations were developed across these domains.

**Conclusions:**

The relative position of individual health care workers within the overall social network in hospitals is associated with influence and agency. As such, power to bring about change is inequitably and socially situated, and subject to specific contexts. The findings of this realist synthesis offer a lens through which to understand social ties in hospitals. The findings can help identify possible strategies for intervention to improve communication and distribution of power, for individual, team and wider multi-professional behavioural change in hospitals.

## Background

The ‘software’ of a health system, that is, the ideas, interests, norms, values, power and relationships between staff, are important to its function, and contribute to the ongoing provision of quality patient care, alongside more tangible ‘hardware’.^1^ However, the social interactions and relational ties between staff in hospitals are often informal and difficult to apprehend.^2^ They are complex, nuanced and fluid, with many of the important interactions for clinical decision making not occurring in the more structured and observable ‘front stage’ (in the presence of patients or carers) or ‘planned back stage’ exchanges. Instead they take place in the ‘ad hoc back stage’ world of corridors and other opportunistic spaces that offer the chance for focused inter-professional exchange, in relative privacy.^3^ Capturing these important informal and ad hoc interactions in a meaningful way presents a challenge, with methods such as social network analysis providing one way of doing so.^4^

The social ties that health care workers form with one another are an important and under-researched component in understanding collective behaviours of staff and ‘practical norms’ in hospitals.^5^ Social ties and resultant social networks were found to reinforce resistant behaviours, or conversely can foster changes in collective behaviours and new ways of thinking.^4,6–9^ Understanding the roles of social networks in staff behavioural change, and the ability to apply such understanding in hospitals, is therefore an essential step in improving, maintaining and disseminating quality of patient care.

There are established behavioural change theories within health care,^10–13^ but a holistic conceptual understanding of staff social ties in hospitals would offer an additional lens within this theoretical space, and an accessible ‘nuts and bolts understanding’ for practical application in quality improvement interventions.

In this paper, we used realist synthesis to explore how, why, for whom, to what extent and in what context the social ties of staff within a hospital influence quality of service delivery, including quality improvement. We used data from social network analysis studies in hospital settings to explore the different social ties and influence between individuals in the same network.^4,9^ Through realist synthesis and the development of abstracted theory, this paper seeks to understand what is common and can be applied to practice. ‘Sets of ideas’ or theory can offer transferability of findings between complex settings, where traditional quantitative approaches have struggled to offer solutions.^14^ This synthesis aims to develop a theoretical lens from the available evidence.

The analysis presented in this paper was part of a larger body of work on quality improvement in neonatal units in Kenya. Neonatal units are examples of multi-professional settings where care is provided to high numbers of complex patients, and where resilient behavioural norms and professional silos have been observed.^15^ Strategies to improve communication ties between neonatologists and senior neonatal nurses have already been implemented across different hospitals,^16^ but quality improvement through social networks is less well explored and this study seeks to contribute to filling this evidence gap in order to guide future empirical work in Kenya.

## Methods

We used realist synthesis^14,17–19^ and followed reporting guidance according to the RAMESES standards.^20^ In brief, realist research produces explanatory theory bound by common language and using an explicit approach (i.e. context-mechanism-outcome configuration, CMOC). It seeks to explain causation, thereby identifying intervention strategies likely to effect change, based on targeted manipulation of the identified CMOC.^19^ Context refers to a specific aspect of a setting which triggers a specific mechanism, which in turn refers to a latent, often invisible property of a person, object, or institution, which is triggered or activated by exposure to a specific context and causes a specific outcome to occur.^21^

We used an initial programme theory, which was informed by established theory and practical experiences of working in a hospital setting, to develop the search strategy (see Online Supplement, Section A). A specialist health librarian (EH) helped to develop the search strategy and select relevant databases (Online Supplement, B), which were searched from inception to 17–18 December 2018 (EH) without language or date restrictions.

All retrieved citations were screened by CB, with 10% also screened independently by AD to check for systematic error in the inclusion or exclusion of documents. Titles and abstracts were screened, followed by full texts of relevant documents, against inclusion and exclusion criteria (Table 1). If two or more documents related to the same study, all additional reports were included only if novel data from the study were reported. Full included documents were imported into NVivo v12.0 for data extraction. Data of relevance to the research question were extracted and coded. Codes were assigned to sections of text within any included document, and 10% of refined iterative coding (stage 3 below) was checked for consistency by AD, with feedback given to CB; further stages of analyses were discussed with AD and the rest of the research team. The research team had expertise and experience, including realism, health systems research, global health, sociology, ethnography, health information, and clinical practice.

**Table 1.** Inclusion and exclusion criteria.

Inclusion criteria	Exclusion criteria
•Empirical study (i.e. both observation and intervention designs)	•No empirical data collection
•Must use a form of social networks analysis in methodology	•No use of social networks analysis (SNA) in methodology
•Hospital setting	•Community, primary care, public health, or health policy setting
•Primary focus on social networks of hospital staff (i.e. clinical and non-clinical staff)	•Primary focus on social networks of patients, carers, relatives, lay health workers or students
	•Focus on online social networking
	•Studies using insurance claims-based data

Data extraction and analysis were undertaken concurrently, using an immersive, iterative and refining approach.^19,22^ Iterative cycles were undertaken to help visualise, make sense of and configure the data, seeking to further refine context-mechanism-outcome-configurations using repeated cycles of analysis (Online Supplement, C). We sought for semi-regular outcome patterns in the data^21^ and developed CMOCs from data contained within and across included documents in several stages: (1) We conducted iterative coding for 25 documents, coding relevant data using descriptive terms deemed to reflect the major theme of the data as interpreted by CB. Novel codes were created by CB whenever relevant data did not fit with an existing code derived from the initial programme theory. We did not set a limit for the number of new codes. (2) We reviewed and refined nodes by re-reading coded data and used manual visualisation/mind-mapping and summarising of data under each node on flip chart paper. Duplicated or equivalent meanings of codes were combined or collapsed, and similar codes were organised into groups along major themes identified from data extracted from the included documents, thus creating new refined nodes. (3) We used refined nodes alongside continued iterative coding to code 34 further reports. (4) Identified nodes were exported into Microsoft Word, and sorted manually in Word documents into more detailed sub-nodes to expand emerging thematic granularity in the data. (5) We followed this step by visualisation and summarising of coded data, using a series of large whiteboards for each higher-level node, to identify explanatory pathways from data within each code. This step sought to begin to move away from thematic coding towards constructing theoretical explanatory units from the data, which would be used as new codes. (6) Identified explanatory pathways were subsequently converted into abstracted explanatory statements from each higher-level node and transcribed from the whiteboards into Word. These explanatory statements were written in prose, allowing greater clarity in identification and absorption of all relevant supporting data. (7) All explanatory statements from the higher-level nodes were then integrated by manually cutting and sticking printed colour-coded text from transcribed statements onto a large piece of flip chart paper. This allowed any duplication and iteration of a common pathway to be identified, and combined. Identified statements could be visually sorted and ordered into domains in relation to one another, thus contributing to programme theory development. (8) From the processes described in steps 5–7, higher-level nodes were then created and further refined, and these refined nodes were used to subsequently code the data from the remaining documents. (9) Newly coded data were used to refine, expand and enrich existing explanatory statements. (10) We constructed CMOCs as written sentences with clear identification of the context, mechanism and outcome in each, to articulate the granularity and detail of explanatory statements and define these as distinct explanatory units of middle-range theory. (11) CMOCs were further refined by discussion between authors, and by open discussion of methodology and emerging findings as part of a realist tutorial group, attended by CB and GW. (12) Data extracted from all included documents were finally re-read and assigned to each CMOC by CB, providing a further check that refined CMOCs were indeed representative of and arising from the data, and were sufficiently supported by evidence (Online Supplement, D). A final step used refined CMOCs to build realist programme theory.

Authors drew on their personal experience when discussing and interpreting findings from the literature, including clinical work in hospitals and primary care, clinical and academic work in the health systems in the UK and sub-Saharan Africa (particularly Kenya and Sierra Leone), and academic expertise including realism, medical sociology and health care information. Data interpretation and analysis were informed by feedback from six additional stakeholders from sub-Saharan Africa with relevant content expertise and experience to sense-check emerging explanations of phenomena within the programme theory (1, Sierra Leone, 5, Kenya), over a period of two years. Of these, four stakeholders (1, Sierra Leone, 3, Kenya) engaged in formal discussions with CB, providing feedback at different stages of the review. CMOCs were derived from the data, mostly from high income settings, and potential relevance to Kenya was considered as part of the larger project. Interim CMOCs were also discussed as part of a student realism seminar group (CB).

## Results

The search retrieved 10,910 citations after deduplication, of which 365 underwent full-text assessment for inclusion. Of these, 75 documents were included in the review (Online Supplement, E). Included documents were from high income countries (92%, 69/75) or upper-middle income countries (8%, 6/75) (Online Supplement, F). About one quarter of studies centred on doctors (17/75), 17% on nurses (13/75) and the remaining on more than one professional group (45/75). Studies examined different types of communication ties from which to collect social network data, including general professional communication ties, medication advice ties and problem solving ties.

Box 1 presents the programme theory developed from the literature. It was developed from 35 unique CMOCs, which are given in Table 2. An expanded version of Table 2 is available in the Online Supplement, G, with illustrative quotes from the literature. The CMOCs are organised in four overarching theoretical domains that we identified: (1) social groups, (2) hierarchy, (3) bridging distance and (4) discourse.

**Table 2.** Context-mechanism-outcome configurations (CMOCs).

Domain 1: *Social Groups*. Close social ties formed with others, based on identity and personal view of the world (representation), in relation to the individual	
CMOC1	When health care workers share a common identity (C), they will be more likely to trust one another (O), as they share an underlying representation (M)
CMOC2a	When health care workers share a representation (C), they will preferentially communicate with one another (M) leading to insular communication (O)
CMOC2b	When health care workers share a representation (C), outsiders are easily identified (M), leading to communication boundaries (O)
CMOC3	When a health care worker has an existing reciprocal relationship with a peer (C), they will preferentially seek advice or support from that person (O), because they trust the person (M1) and feel comfortable (M2)
CMOC4	When health care workers share a representation (C), this can lead to redundant information (O1), group-think (O2) and echo (O3), due to insular communication (M)
CMOC5a	When a health care worker is central within a social group (C), their personal behaviours will embody group norms (O) because they will hold tightly to group identity as own (M)
CMOC5b	When a health care worker is peripheral within a social group (C), they may deviate in behaviour from group norms (O1), and oppose/conflict with central members of the group (O2) because they may not wholly identify with the group representation (M)
CMOC6a	When a health care worker is central within a social group (C), they will have greater power over group norms (O), because they have greater influence over group narratives and representation (M)
CMOC6b	When a health care worker is peripheral within a social group (C), they will have little power over group norms (O), because they have little influence over group narratives and representation (M)
CMOC6c	When a health care worker is central within a social group (C1) and has power over material resources (C2), they will have additional power over group norms (O) because they own the distribution of material resources and decision-making authority (M)
CMOC7a	Where a health care worker requires diverse information to undertake their role effectively, for example, senior nurse, junior doctor (C), they will become more central in the workplace information-sharing network (O), because they must frequently seek information from others (M1) and others frequently seek information from them (M)
CMOC7b	When a health care worker is peripheral in the workplace information-sharing network (C), they risk isolation (O), because they are not actively engaged in information-seeking (M1) or information-sharing (M2) with colleagues
CMOC7c	When a health care worker needs legitimate/defensible information (C), they will speak to someone who embodies desirable group norms (O), because the information received will comply with social expectations (M)
Domain 2: *Hierarchy*. Social ties or barriers formed based on hierarchical status of the individual	
CMOC8a	When an individual is senior in the hierarchy (C1), or is a custodian of hierarchical status (C2), they have permission to approach both high and low status colleagues (O) because of understanding of the ‘social rules of operation’ (M)
CMOC8b	When an individual is lower in the chain-of-command/hierarchy (C), they lack permission to approach some colleagues directly so access new/external information through the established chain of command (O1), or through a broker (O2), because of understanding of the ‘social rules of operation’ (M)
CMOC9a	Where hierarchical control is paramount to the functioning of a social group (C) members will be more likely to accept and follow orders from a senior (O) due to concentration of authority (M)
CMOC9b	Where hierarchical control is not paramount to the functioning of a social group (C) members will need to be convinced to change behaviour (O) due to flattened structure and distributed authority (M)
CMOC10a	Where parallel hierarchies are operating within the same workplace (C), permission to approach colleagues outside one’s own hierarchy structure will vary (O), according to the specific authoritative power over the individual and their understanding of the ‘social rules of operation’ between hierarchies (M)
CMOC10b	Where doctors have historically taken the role of leader in health care teams (C), they will assume a position of authority (O), because of their understanding of the ‘social rules of operation’ (M)
CMOC11a	When there is a hierarchical structure (C), there is a risk of fragmentation into sub-groups based on status (O), because individuals identify more closely with peers of the same status (M1) and trust them more (M2)
CMOC11b	When an individual is higher up in the chain-of-command/hierarchy (C), they will preferentially communicate with other higher status staff (O), because they feel higher status colleagues will better understand their scope of work (M1), and will hold more useful information (M2)
CMOC11c	When an individual is lower in the chain-of-command/hierarchy (C), they will only expect specific types of support from colleagues of higher status (O), because of their understanding of the ‘social rules of operation’ (M1) and because they feel more comfortable with peers for usual support (M2)
CMOC12a	When an individual is higher up in the chain-of-command/hierarchy (C), they will preferentially communicate with other higher status staff (O), because they will further enhance their own status/power by association (M)
CMOC12b	When an individual is higher up in the chain-of-command/hierarchy (C), they will have power over information (O) by controlling the information and resources disseminated to low status colleagues (M)
Domain 3: *Bridging distance*. The ways in which an individual can be encouraged to communicate with others with whom they do not have a close social tie	
CMOC13	When a health care worker observes a non-peer colleague displaying they are being ‘careful’ (C), they will exchange required information with this colleague (O), because they feel trusting towards the colleague (M1) and feel comfortable approaching them (M2)
CMOC14	When a health care worker has a longstanding or multiplex relationship with a non-peer colleague (C), they will approach that colleague as a ‘go-to person’ (O), because of mutual understanding (M1), and trust (M2)
CMOC15	When a health worker needs information urgently (C), they will select a colleague from those who are physically present (O), because they will receive an immediate response (M)
CMOC16	When a health care worker can speak to a colleague face-to-face (C), information exchange will be enhanced (O), because they can better read and respond to verbal and non-verbal cues (M1) and is more confident that they will be understood (M2)
CMOC17	Information can be exchanged between disconnected groups (O), when there is an individual who can act as a broker (C), because they communicate with members of both groups (M)
Domain 4: *Discourse*. The totality of the different ways of seeing the world, both content but also how each relates to the other and is communicated, in terms of overall dominance and power	
CMOC18a	The pattern of ongoing exchange and negotiation between social groups in the hospital workforce (C) creates and maintains (O1) or challenges (O2) the dominant discourse in a hospital, by demonstrating and exerting the relative power of different group representations within the hospital workforce (M)
CMOC18b	For health care workers in an organisation, its rituals and norms (C) construct what an individual perceives is acceptable in the workplace (O), through the demonstration of social expectations and etiquettes (M)
CMOC19a	When a health care worker has to do or say something at work (C), they keep to within a ‘bandwidth’ of actions they perceive to be ‘accepted’ or ‘permitted’ (O), based on their understanding of what the dominant discourse is within a hospital and their relative personal position based on identity and hierarchical status (M)
CMOC19b	The perceived values of an organisation (C) facilitate the creation of status differentials within the workplace (O) because individuals benchmark their activities and identities against how much they embody these values (M)
CMOC20a	Where formal communication processes in hospitals are dysfunctional/unreliable (C), make-shift processes of informal information exchange dependent on social connections result (O) because individuals have to compensate to acquire necessary information (M)
CMOC20b	When informal information exchange becomes the norm in a hospital (C), access to information by specific individuals and groups is inherently skewed (O), because it will depend on how socially connected the individual or group is (M)

**Box 1. Programme Theory**

Hospital staff prefer to communicate with colleagues who are similar to themselves, and with whom they share trust. However, this can create boundaries, silos and redundant information within pockets of the workforce, and different behavioural norms adopted by members of different groups, dominated by influential individuals. Tacit hierarchical rules also determine communication with others in the workplace based on status, forming a landscape of differential access to information and others. Fragmentation between different status groups can occur, and space for status-enhancing behaviours is created. Workplace silos and status boundaries can be bridged in the presence of trust, confidence and mutual understanding, or where need is urgent and immediate. The organisation can actively comply with or challenge social boundaries within the workforce by the formal processes endorsed, which allow day-to-day performances of relative power, based on identity and hierarchical status. Attempts to manage the discourse, and the effectiveness of formal workplace communication processes, act to construct or disassemble social boundaries in hospitals. Connections between staff influence access to information and other forms of capital, behavioural norms and perceived agency. Ties are continuously shaped and as such are amenable to intervention. The capacity of these connections and the overall structure of the workplace social network are vital to delivering quality patient care.

### Social groups

Staff form or discover their own identity as they seek to place themselves relative to others within the social landscape of the hospital, wherein *identity work* (the ongoing process of forming and maintaining a sense of self) occurs.^23^ For example, due to the long-established, rooted, tightly-held, defended and sometimes hidden objects which comprise a profession and its associated representations, group membership is strong and barriers are often very distinct between different professions.^6^ People tend to spend time and communicate with people who are like them in some way (*homophily)*.^4^ As such, social groups form whose members share a way of seeing the world, or *representation*.^24^ This builds trust between group members (Table 2, CMOC1).

Group representation, that is, a way of seeing, telling and interpreting the world, gives content, structure and boundaries to the social identity of the group and its members, and meaning to purpose and behaviours. It is socially communicated by means of narratives.^25^ Sense making of day-to-day experiences uses ordering and labelling to construct a continuous underlying explanatory narrative to life.^26^ As an individual health care worker, or a group of workers experience some action or event (either first-hand, or through the observed experiences or reports of social peers), sense is made of the experience by measuring it against the group narrative and representation.^26^ Therefore, those belonging to the same social group are more approachable to one another than outsiders (CMOC2a)**,** whereas those who do not affiliate with the group’s representation^24^ are easily identified as ‘other’. Assumptions and judgements are made regarding values, background and behaviours of the ‘other’.^27^ In the hospital workplace, othering can occur between health care workers and patients,^27,28^ and between different professions.^28^ The labels, judgements and assumptions that are made about the ‘other’ can lead to communication barriers with those colleagues who are outside one’s own immediate social group. Over time, individuals become increasingly easily identified as part of the group, or outsiders (CMOC2b)**.**

Health care workers will preferentially communicate with peers with whom they have an existing reciprocal relationship, due to familiarity and trust. Reciprocation is associated with commonality and work tasks which require interdependence of colleagues, for example, administering drugs and manual handling of patients. Reciprocating peers are more likely to form strong ties with one another. As communication and reciprocation increases, trust is further enhanced.^29^

Communication within a social group becomes more frequent and more insular over time, enhancing homophily of group members and hence trust and preferential communication.^4^ While the consequences may be positive in some instances, an unchallenged uniform outlook can develop and intensify over time. Insular communication can thus risk redundancy of information within the group, where individuals cannot access novel information and knowledge.^4^ A strong preference to communicate only with those who are like oneself can lead to unchallenged collective convictions, and social-tailoring of information acting to strengthen existing outlook and tacit knowledge, that is, groupthink,^30,31^ and echo^30,32^ (CMOC4).

Within a social group, individuals who most strongly identify with that group’s representation, and act to defend its position in the wider field and discourse,^24^ are usually most central to the process of information exchange within the group. As such, central members^4^ model the group’s behavioural norms to its members, and are more influential over the behaviours of other group members than those in peripheral positions (CMOC5a). When an individual is less invested in mainstream group identity, their behaviours may deviate from the expected group norms, either due to oversight or by intentional action. The opposition by central powerful members to such deviance from group norms by peripheral members can lead to conflict and even potential risks or threats (CMOC5b).

Group representations^24^ and narratives are protected, shaped and propagated by those most central within the group with the most social power. Behavioural norms are established from group narratives, and group members experience social pressure to conform to expected norms (CMOC6a). Conversely, those in the group who are peripheral have little influence on the formation and stewardship of group representations and norms, but are expected to conform or face disapproval or remediation (CMOC6b). Material resources are more often controlled by central members, who are also more likely to be involved in decision-making within and on behalf of the group. The group centre-periphery structure is therefore maintained by the distribution of both physical and social capital within the social group and power can be enhanced when formal authority is aligned with social centrality (CMOC6c).

The entire multidisciplinary health care workforce also displays a macro centre-periphery structure in hospitals. In this macro centre-periphery structure, centrality of individuals is linked to job activity and status. In many hospital wards, junior doctors, senior nurses and some ward-based allied health professionals (e.g. ward pharmacist) occupy central positions, even though in the example of junior doctors the specific individuals filling these roles often frequently rotate (CMOC7a). Staff on the periphery of the macro communication network in the hospital workplace (e.g. senior doctors, junior nurses) can become isolated from information flowing from and between more central members. Fragmentation of the macro communication network can be exacerbated where ward activities are undertaken in isolation from other staff, or where staff are separated by shifts or physical proximity without adequate information sharing (CMOC7b). Since central individuals are seen to model and influence behaviours, advice or information received from a central colleague will have legitimacy. Indeed, seeking advice from a central colleague can hence be justified to others (CMOC7c).

### Hierarchy

Health care workers operate within different hierarchies, each with a set of tacit social rules by which the workforce is expected to abide in daily workplace interactions. Such rules show respect and deference to status, and maintain lines of command. Those higher up in the hierarchy are permitted to communicate freely with colleagues of their choosing. By virtue of their ‘custodial’ hierarchical status^33 (p. 853)^ junior doctors also have direct access to colleagues who are of both higher and lower status to themselves (CMOC8a). Hierarchical rules rarely permit direct communication by those lower down with those higher up the chain of command, instead risking offense or discipline. Lower status staff are therefore limited to communication with peers, direct seniors, or brokers (individuals positioned between two otherwise disconnected groups).^4,34,35^ Information and knowledge accessible to such staff is therefore limited, and risks lower status individuals becoming disenfranchised if information sharing is perceived to be inadequate (CMOC8b).

Hierarchical rules in hospitals provide a backdrop against which the behavioural norms of staff operate. The strength of influence of hierarchy on norms, compared with other factors (e.g. social groups), will differ by setting and by social group. In some instances, hierarchical control may be a principal determinant of norms, whereas in others hierarchy may play less of a role (CMOC9a and 9b). A health care worker’s own professional hierarchy will also dictate with whom they are permitted to communicate from other professions. As such, profession-specific hierarchies not only create the rules of communication within professions, but also determine rules for communication with colleagues of other professions (CMOC10a). Social rules based on historical hierarchies between different professions (i.e. doctors historically senior to nurses) are also significant in some settings (CMOC10b).

A hierarchy can escalate differentiation and power imbalance without sensitive leadership and management skills and awareness, and risks alienation of lower status health care workers from those of higher status, and vice versa. This can occur when perceived commonality and mutual understanding across hierarchical bounds is lost and replaced by difference, distance, division and even conflict (CMOC11a). As well as line-management responsibilities over more junior staff, senior staff often undertake greater problem-solving and resource allocation work, compared to their juniors, who undertake a greater proportion of activity-driven work.^36^ The workforce can therefore become internally differentiated by its dichotomous scope of work, by seniority (CMOC11b). Junior staff differentiate themselves from their seniors, with peer relationships at a common hierarchical level often preferred (e.g. among junior doctors).^37^ Consequently, junior staff can become increasingly functionally independent of seniors, albeit for specific purposes of communication, legitimisation or support.^38^ Peers within the same status group learn to primarily depend on one another for support and information, leading to redundancy and loss of information transfer between different levels of seniority (CMOC11c).

If an individual exchanges information or builds a relationship with a high-status colleague, their own status will increase by association.^4^ In addition, the individual may gain access to other forms of capital (e.g. material resources) from the high-status colleague, which will further increase own status.^7,39^ (CMOC12a). When there is a strong chain of command structure, those lower down in the hierarchy will be dependent on their higher status colleagues for information and resources. Higher status staff therefore have power over the acquisition of capital by their lower status colleagues, by determining how information and resources are distributed (CMOC12b).

### Bridging distance

The workplace allows health care workers to observe behaviours and attitudes of colleagues, including those not part of their immediate social peer group. They will use this learning to choose which non-peer colleagues to trust and feel comfortable to approach, that is, bridging distance, for exchange of information, based on the personal communication skills and behaviours they observe, that is, are they being ‘careful’.^40^ In addition to personal observations, the stories and accounts of others, particularly those of trusted peers, will allow an individual to assess whether to trust a non-peer colleague (CMOC13). Some health care workers build up relationships with non-peer colleagues over time, or through multiplex ties (when two individuals are connected by more than one type of interaction).^4^ Multiplex ties can form, for example, as the product of previous postings, or through knowing an individual through another means (e.g. being on same committee). If a health care worker works for a long time in the same place, understanding and commonality will accrue through collective experience and observation. The worker will become familiar with areas of expertise and resource capital of specific colleagues, and will understand how best to negotiate access to required information or resources (CMOC14).^41^

When information is required urgently, a health care worker will preferentially seek information or support from a colleague who is physically present to increase the immediacy at which their need is met. Colleagues who are physically present in the workplace are thus more likely to be approached by others (CMOC15). Face-to-face communication allows picking up on non-verbal communication cues and, as it is interactive,^42^ clarification of misunderstandings and reciprocal information sharing. Alternative forms of communication might include telephone, email or written notes (CMOC16).

Brokerage of information occurs when an individual who is independently accepted by two otherwise disconnected groups, transmits and mediates information between these groups.^4,34,35^ Acceptance as a broker can be gained by merit of interpersonal skills, other desirable attributes, for example, reputation for being hard-working, cross-cutting scope of work activities, or alternatively by a formal role, such as senior nurse. Information transferred by a broker is usually advantageous to the group and novel, however the broker must mediate information, which may lead to incomplete or selective information sharing. Significantly, the broker is able to ‘translate’ the information for the specific and different ‘languages’ of both groups. The position of broker is fundamentally tied to power and status (CMOC17).^4,34,35^

### Discourse

Discourse is not only the summative but also the relational landscape of representations held within a hospital’s entire workforce, both present and past.^24^ Through the process of exchange over time, communication is dominated and characterised by certain representations.^43^ Discourse maintains or challenges the dominant status quo.^44^ By ordering the hospital workforce in relation to one another by means of representations, it is possible to understand the relative power of others compared to oneself, which can be important in framing and interpreting one’s ‘life space and the world at large’^45 (p. 214)^ Similarly, the theory of fields describes a ‘struggle’^46 (p. 109)^ between different social groups, with powerful groups seeking to maintain their advantage, and challengers seeking opportunities to create ‘a new order’ (CMOC18a).^46 (p. 118)^ Rituals and norms communicate discourse, by embodying ‘how things are done around here’.^47 (p. 1)^ Rituals and norms (e.g. ward rounds and handovers) communicate tacit power structures in the hospital workplace, sustaining workplace power structures by defining and reinforcing expectations and social rules (or ‘etiquettes’) (CMOC18b).

Discourse places people within the social whole, based on relative dominance of their held representation. However, representations also shape the discourse.^7,24^ Location and power of a specific representation within the discourse determines the agency of its members, through a process of comparison with others. Resultant agency is compounded by hierarchical status (CMOC19a). How a health care worker perceives organisational values is dependent on what is observed (either directly or through reputation from others) and then interpreted, through the lens of the representation held by their social group.^24^ If an individual feels aligned with organisational values, they will assume greater legitimacy and confidence in the workplace, but they will not if they do not feel aligned. For example, some health care roles will attract greater esteem at institutional level, depending on their degree of embodiment of perceived organisational values. Clinical roles, particularly those associated with decision-making and cure (i.e. doctors), are often more highly esteemed. Conversely, roles such as cleaning, while of great importance, are less esteemed. Distribution of social capital and power in the workplace, and the identity work of individuals and different occupational groups within the hospital, are influenced as they act to continually construct a sense of self within the health care workplace (CMOC19b).^48^

Where formal communication processes are inadequate, socially determined exchanges become essential for acquiring necessary workplace information. With time, informal exchanges can bypass or even replace formal processes, leading to their further deterioration (CMOC20a). While socially determined exchanges might be preferable for well-connected individuals, they inherently construct systematic inequalities in access to information. For those more peripheral or of lower hierarchical status, reliable information can become very difficult to access. This leads to skewed information access in the hospital workplace, determined by the social position of the individual worker as reflected in the theory of embeddedness (CMOC20b).^29^

## Discussion

This paper used realist synthesis of social network analysis studies to identify and understand social ties of staff in hospitals that act to influence behaviour. We started a process of developing middle-range theories, and propose that a health care worker’s social position in a hospital is influenced by 35 explanatory pathways (CMOCs) across four overarching theoretical domains: social group, hierarchy, bridging distance and discourse. These domains should be further refined by ongoing theory-building and do not represent a final set of CMOCs.^17^ In essence, relative social position influences an individual’s capital, influence, power and agency in the workplace. The social ties that exist between hospital staff are therefore fundamental to the targeting and success of quality improvement activities and behavioural change programmes.

### Social ties of staff and quality improvement

Our findings offer an alternative lens through which to view social concepts associated with interpersonal communication, such as teamwork. For example, it is known that positive teamwork and good coordination within the health care team can act as a resource for team members in the provision of safe patient care, as well as enhancing staff wellbeing in the workplace, which in turn further improves patient safety and positive team working.^49^ Conversely, a dysfunctional team environment does not provide a safety resource for health care staff, but can put additional strain on the individual over and above their existing workload, negatively impacting on patient safety.^49^ We begin to develop explanations about how, why, for whom, to what extent and in what context the informal communication networks of health care workers form and might be influenced, thereby offering theory from which interventions aiming to effect aspects of ‘software’^1^ such as teamwork, can draw.

Quality patient care is synergistic and associated with multidimensional features, including staff social aspects. Liberati et al.^50^ identified seven features of safety, including teamwork, cooperation and positive working relationships, respectful relationships and reinforcing of safe, ethical and respectful behaviours. Our analysis has developed a way to identify relational concepts and begins to develop an explanation as to their importance, as well as linking concepts, adding to the existing evidence base. Specifically, our findings support the established theoretical importance of social ties in maintaining behavioural norms in the hospital setting,^4,5^ and in influencing behavioural change of individuals and groups of health care workers.^4,7,8^ We offer conceptual theory as a practical lens for understanding key social influences in these complex multi-disciplinary settings. The identified context-mechanism-outcome configurations offer an explanatory access-point to the concepts of how social ties influence action, which can inform interventions for quality improvement in hospitals. By explicitly mapping a causal pathway, context-mechanism-outcome configurations (CMOCs) are useful for identifying the possible reasons for workforce behavioural norms in a given setting, and what strategies might enhance adoption of new behaviours and quality improvement. Through abstraction, the conceptual becomes more amenable to practical application across unique hospital settings.^14,19^ Communication influences and distribution of power can potentially be identified. Barriers can be mitigated through targeted theory-driven interventions, and quality improved in different hospitals.

### Strengths and limitations

Strengths of this synthesis included: a comprehensive search strategy with no language or date restriction, inclusion of documents studying different contents of ties within the workplace (e.g. from friendship, to medication advice ties), refining of analysis and interpretation in discussion with stakeholders, realist researchers, among authors with broad collective experience, and analogy and consilience with existing theory. However, the majority of studies were from high income countries and we sought to mitigate this by involving stakeholders from low income countries to provide feedback at different stages in the synthesis. Still, our findings should be further refined by data from low and middle-income countries to help understand issues that may be specific to low-resource settings. We also only carried out one search and did not update it because we judged our approach to provide sufficient relevant data.

Further work should explore the utility of the synthesis presented in this study to develop a theoretical framework for capturing learning from diverse quality improvement initiatives across different hospital settings, to enable transferability of learning by identification of common explanatory threads.

## Conclusions

Health care workers are socially positioned relative to one another within the hospital workforce, which fundamentally determines their access to information, support from others and the possible actions available to them. Members of the workforce do not possess equal capital, or equal agency for change. Power to bring about collective behavioural change in the workplace is inequitable, socially situated and subject to specific identified contexts. Realist synthesis was useful for developing granular theory to understand social ties in hospital settings and the significance of these for behaviour. The findings can help identify areas for intervention to improve communication and distribution of influence and power, and thereby support behavioural change and quality improvement initiatives in hospitals.

## Supplemental Material

sj-pdf-1-hsr-10.1177_13558196221076699 – Supplemental Material for The social networks of hospital staff: A realist synthesis Supplemental Material, sj-pdf-1-hsr-10.1177_13558196221076699 for The social networks of hospital staff: A realist synthesis by Claire Blacklock, Amy Darwin, Mike English, Jacob McKnight, Lisa Hinton, Elinor Harriss, Geoff Wong in Journal of Health Services Research & Policy
