# Supplementary material for: The social networks of hospital staff: A realist synthesis
Source: J Health Serv Res Policy. 2022 May 5;27(3):242–52. doi: 10.1177/13558196221076699 (PMC9277319; doi:10.1177/13558196221076699)
Supplement: sj-pdf-1-hsr-10.1177_13558196221076699 – Supplemental Material for The social networks of hospital staff: A realist synthesis [file sj-pdf-1-hsr-10.1177_13558196221076699.pdf]

Journal of Health Services Research & Policy
The social networks of hospital staff: a realist synthesis
Claire Blacklock, Amy Darwin, Mike English, Jacob McKnight, Lisa Hinton, Elinor Harriss and Geoff Wong

Online Supplement

(A) Initial Programme Theory

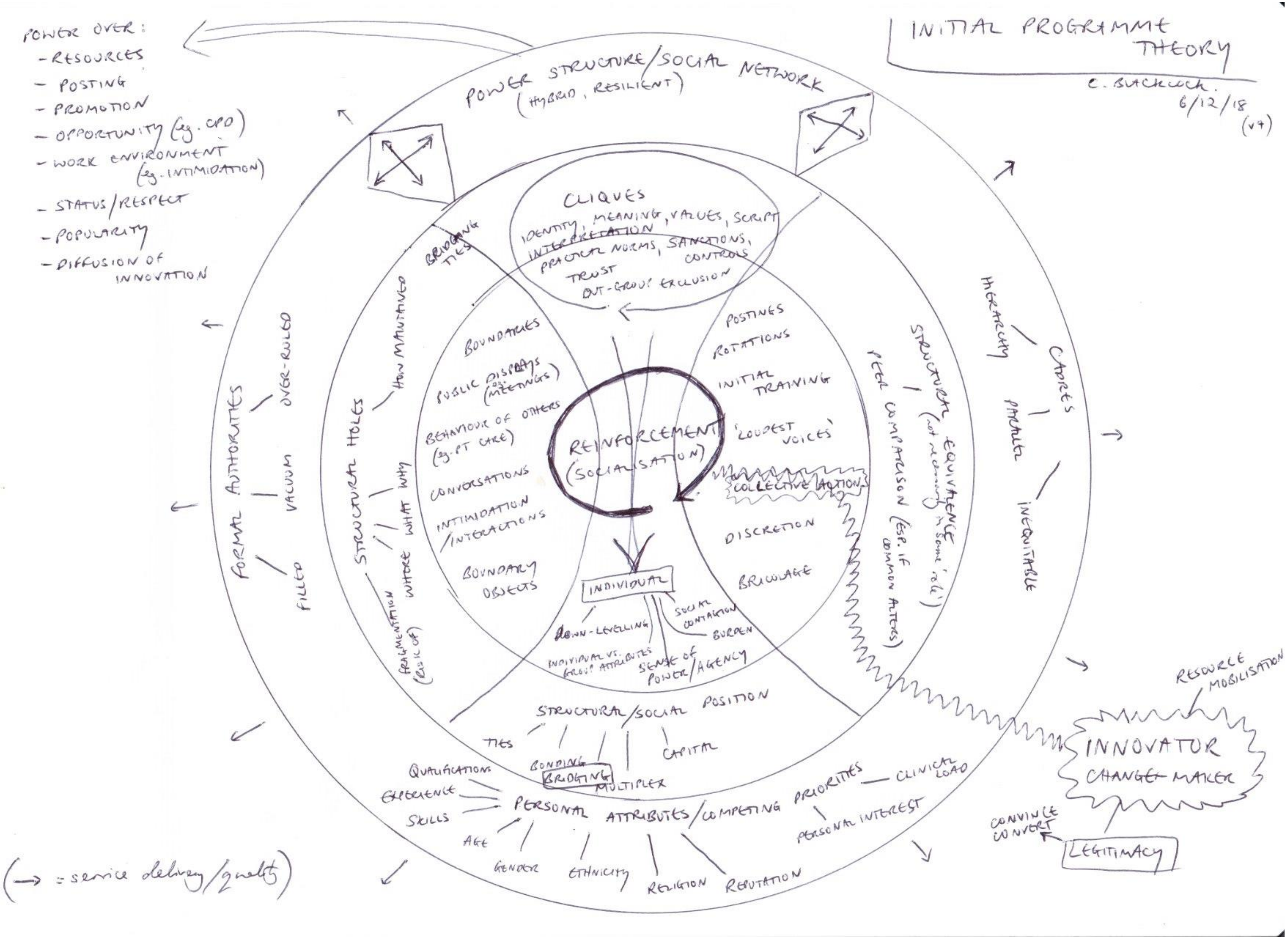

(B) Search terms

Participants/population	Intervention(s)/exposure(s)	Main outcome(s)
"Personnel, Hospital"[MeSH Terms]) OR ("Health personnel"[MeSH Terms] AND "Hospitals"[MeSH Terms]) OR staff[Title/Abstract]) OR surgeon*[Title/Abstract]) OR midwi*[Title/Abstract]) OR nurs*[Title/Abstract]) OR keeper*[Title/Abstract]) OR porter*[Title/Abstract]) OR supervisor*[Title/Abstract]) OR pharmacist*[Title/Abstract]) OR doctor*[Title/Abstract]) OR physician*[Title/Abstract]) OR manager*[Title/Abstract]) OR leader*[Title/Abstract]) OR technician*[Title/Abstract]) OR therapist*[Title/Abstract]) OR physiotherapist*[Title/Abstract]) OR aide*[Title/Abstract]) OR assistant*[Title/Abstract]) OR officer*[Title/Abstract])) OR administrator*[Title/Abstract])	SNA[Title/Abstract] OR "social network"[Title/Abstract] OR “social networks”[Title/Abstract]	*No search terms included for outcome*

Example search: Medline (Ovid MEDLINE® Epub Ahead of Print, In-Process & Other Non-Indexed Citations, Ovid MEDLINE® Daily and Ovid MEDLINE®) 1946 to present

Search Strategy:

1 exp Personnel, Hospital/ (87424)
2 exp Health Personnel/ (471901)
3 exp HOSPITALS/ (257382)
4 2 and 3 (30656)
5 (staff or surgeon* or midwi* or nurs* or keeper* or porter* or supervisor* or pharmacist* or doctor* or physician* or manager* or leader* or technician* or therapist* or physiotherapist* or aide* or assistant* or officer* or administrator*).ti,ab. (1295006)
6 1 or 4 or 5 (1339519)
7 (SNA or "social network*").ti,ab. (16703)
8 6 and 7 (2136)

Databases searched

EBSCO CINAHL
Ovid EMBASE
Ovid Global Health
Ovid Medline and MEDLINE In-Process & Other Non-Indexed Citations
Ovid PsycINFO
IDEAS https://ideas.repec.org/
ProQuest Sociological Abstracts
LILACS database via the WHO Global Index Medicus Regional Libraries
Social Science Citation Index (SSCI via Web of Science Core Collection)
Science Citation Index (via Web of Science Core Collection)
ProQuest Econlit
ProQuest Applied Social Sciences Index & Abstracts (ASSIA)
EBSCO Business Source Complete
ProQuest ABI/Inform Global

(C) Data analysis

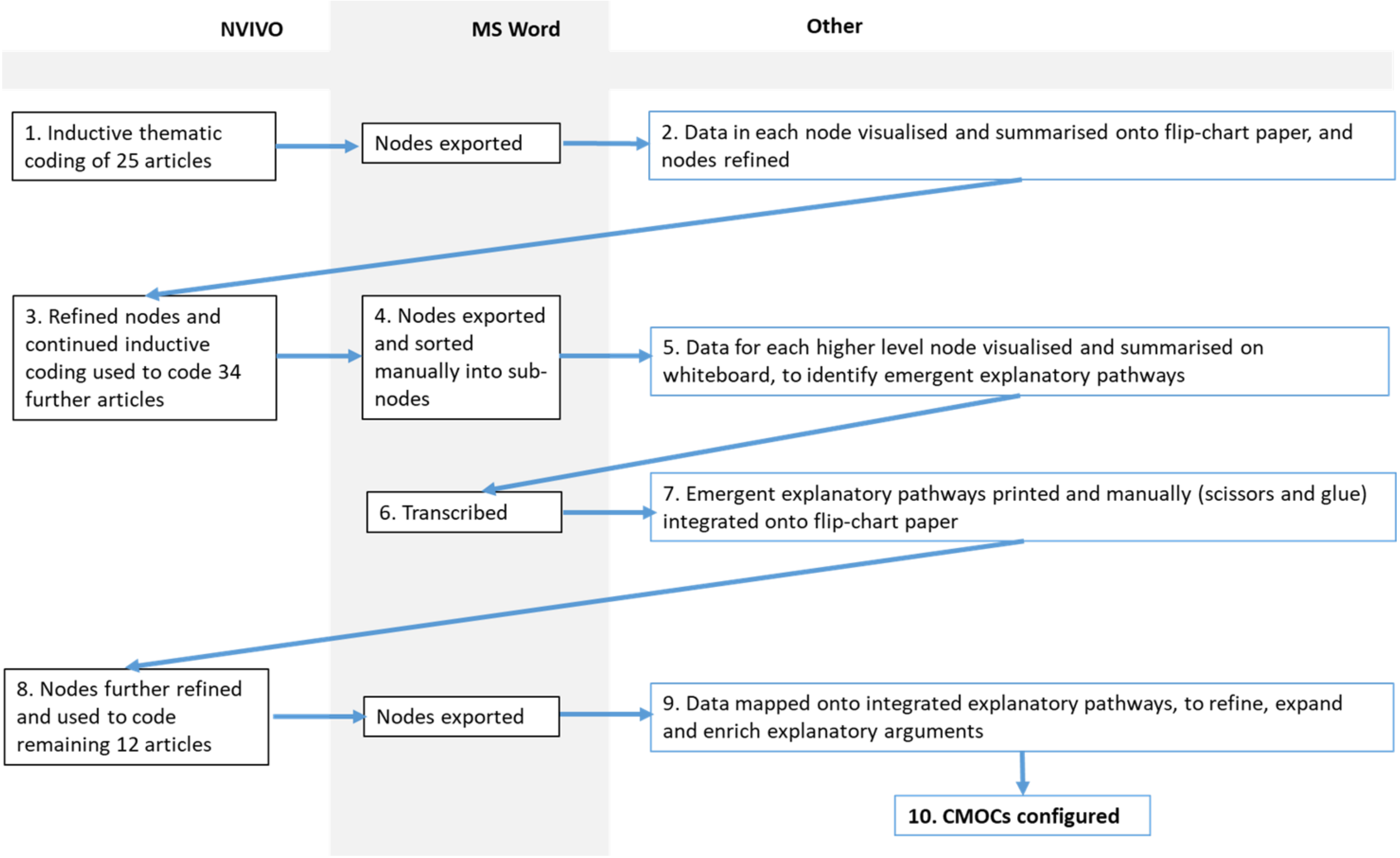

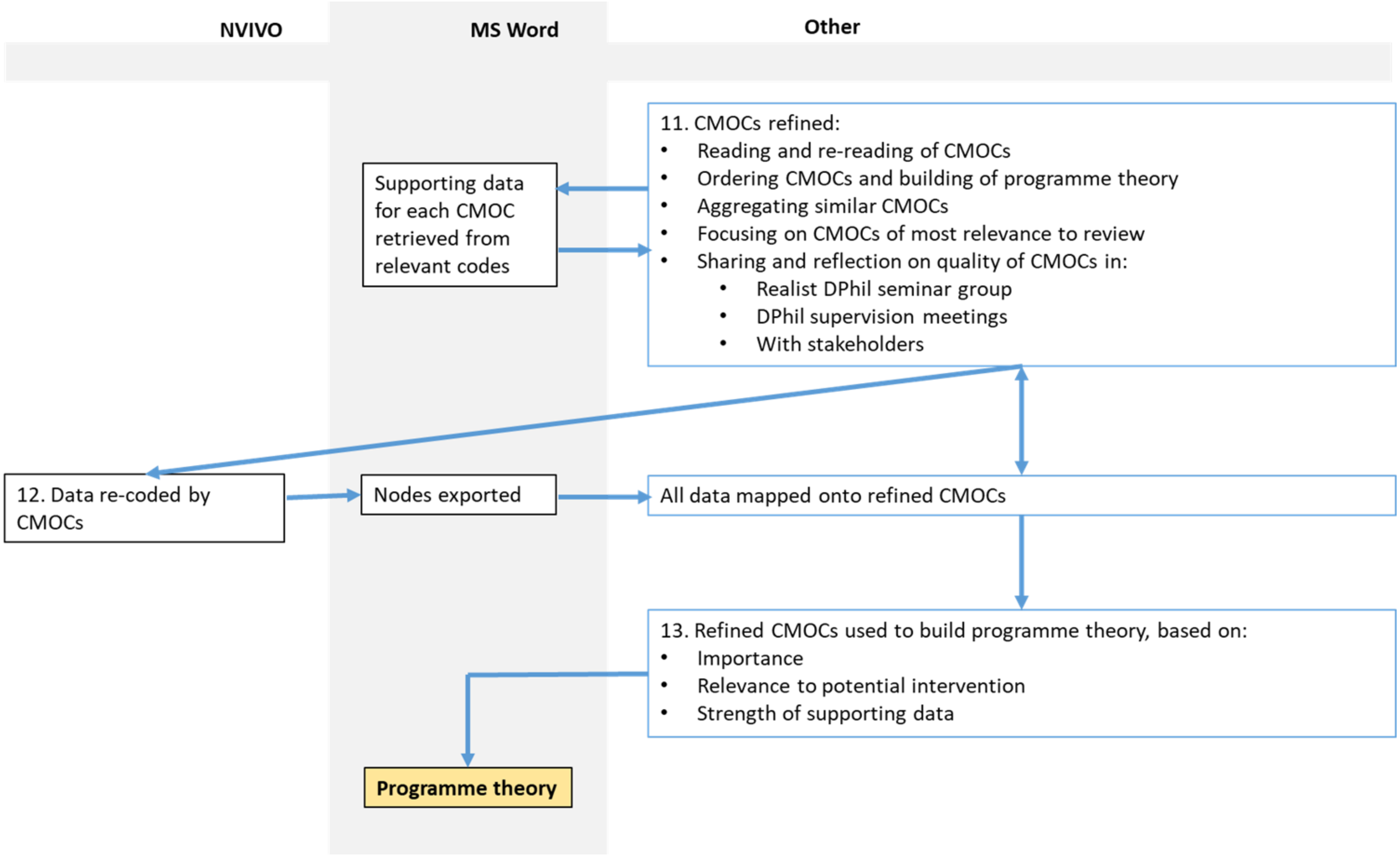
NVIVO
MS Word
Other
11. CMOCs refined:
Reading and re-reading of CMOCs
Ordering CMOCs and building of programme theory
Aggregating similar CMOCs
Focusing on CMOCs of most relevance to review
Sharing and reflection on quality of CMOCs in:
Realist DPhil seminar group
DPhil supervision meetings
With stakeholders
Supporting data for each CMOC retrieved from relevant codes
12. Data re-coded by CMOCs
Nodes exported
All data mapped onto refined CMOCs
13. Refined CMOCs used to build programme theory, based on:
Importance
Relevance to potential intervention
Strength of supporting data
Programme theory

Journal of Health Services Research & Policy
The social networks of hospital staff: a realist synthesis
Claire Blacklock, Amy Darwin, Mike English, Jacob McKnight, Lisa Hinton, Elinor Harriss and Geoff Wong

(D) Supporting data extracted from included documents

(Documents which contributed any amount of supporting data to a specific CMOC are marked '1' in the column denoting the relevant CMOC)

Document	1	2a	2b	3	4	5a	5b	6a	6b	6c	7a	7b	7c	8a	8b	9a	9b	10a	10b	11a	11a	11b	11c	12a	12b	13	14	15	16	17	18a	18b	19a	19b	20a	20b
Anderson 1985	1	1		1	1	1	1	1	1	1			1																		1					
Anderson 1991	1	1	1	1	1	1		1		1				1						1			1					1		1						
Anderson 2011						1	1	1	1	1		1	1																	1						
Atalib 2017																											1				1	1			1	1
Bae 2017		1	1	1																		1	1													
Barrot 2013																																				
Benham Hutchins 2010			1								1			1	1												1	1	1	1	1	1	1	1	1	1
Boyer 2010	1	1	1	1	1						1			1				1	1											1	1		1	1		
Brewer 2018a						1		1		1	1		1												1			1	1	1						
Brewer 2018b				1		1		1		1	1		1												1		1			1						
Cerne 2004																																				
Chan 2017	1	1	1	1				1		1	1	1		1		1		1	1	1	1	1	1		1		1			1	1					
Cott 1997	1	1	1	1	1	1	1	1	1	1	1	1	1	1	1	1		1		1		1		1	1					1	1	1	1	1		
Creswick 2009	1	1	1	1	1						1	1		1	1			1	1	1	1	1			1	1		1		1	1	1	1	1	1	
Creswick 2010	1	1		1	1			1	1	1	1	1		1	1			1		1	1	1		1	1	1	1	1	1	1	1	1			1	
Creswick 2015		1	1						1		1	1			1													1								
Currie 2014																							1							1						
Dauvrin 2015						1	1	1	1	1			1	1											1	1					1	1				
Dauvrin 2017						1	1	1	1	1	1		1												1						1	1	1	1		
Di Vincenzo 2017	1	1		1	1	1		1	1	1	1		1		1		1	1		1		1				1	1	1		1	1	1	1	1	1	1
Effken 2013		1			1		1			1				1	1					1					1					1	1	1			1	
Espinoza 2018	1	1	1	1		1	1			1	1			1	1			1	1							1			1	1	1	1	1	1	1	
Fong 2017								1		1	1		1					1							1	1		1	1	1						
Forsyth 2018						1	1	1	1	1	1	1	1	1	1			1	1	1					1				1		1	1	1	1		
Grace 2015	1			1							1							1	1	1		1			1	1	1			1	1	1	1	1	1	1
Heiligers 2008	1	1		1	1						1			1							1		1				1	1	1	1						

Journal of Health Services Research & Policy

The social networks of hospital staff: a realist synthesis

Claire Blacklock, Amy Darwin, Mike English, Jacob McKnight, Lisa Hinton, Elinor Harriss and Geoff Wong

Document	1	2a	2b	3	4	5a	5b	6a	6b	6c	7a	7b	7c	8a	8b	9a	9b	10a	10b	11a	11a	11b	11c	12a	12b	13	14	15	16	17	18a	18b	19a	19b	20a	20b
Heng 2005											1	1																		1						
Heng 2013		1									1	1																		1	1		1	1		
Hurtado 2018				1																																
Iacopino 2018	1				1																									1						
Iyengar 2011	1			1	1	1	1	1	1	1	1		1													1	1		1	1	1	1	1	1		
Iyengar 2015	1					1	1	1	1	1			1	1				1							1						1	1	1	1		
Janmano 2018					1						1			1	1			1				1	1			1			1	1	1		1	1		
Jippes 2013						1	1	1	1	1	1	1	1																				1			
Jippes 2010	1	1																								1				1	1	1	1	1		
Landim 2010						1	1	1	1	1			1	1	1			1				1	1		1		1			1	1		1	1		
Lindberg 2013	1					1	1	1	1	1	1		1	1	1											1			1	1	1	1	1	1		
Llupia 2016	1			1										1	1								1		1				1			1	1			
Lurie2009																																				
MacPhee 2000	1	1		1										1	1					1			1	1		1		1	1	1	1	1	1	1		
MacPhee 2002	1	1	1	1										1	1	1				1		1	1		1		1		1		1	1	1	1		
Marques-Sanchez 2018	1	1		1												1	1	1	1	1		1					1			1	1	1	1	1		
Mascia 2011a	1	1	1	1	1			1									1						1						1	1	1	1	1	1		
Mascia 2011b	1	1	1	1	1	1	1	1	1	1			1	1			1	1	1			1	1		1		1			1	1	1	1	1	1	1
Mascia 2013	1	1	1	1	1	1	1	1	1	1	1	1	1	1	1		1			1		1		1	1					1	1	1	1	1	1	
Mascia 2015	1	1	1	1		1							1				1			1		1				1	1			1	1	1	1	1		
McCurdie 2018				1						1	1	1		1	1	1		1							1		1	1		1	1	1	1	1	1	1
Meltzer 2010	1	1	1		1	1	1	1	1	1	1		1												1					1	1		1	1		
Menchick 2010						1	1	1	1	1	1	1	1													1					1	1	1	1		
Myers 2016				1																							1									
Pappas 2003	1	1		1	1			1		1	1											1								1	1	1	1	1		
Pappas 2004								1		1															1		1			1	1					
Pappas 2007						1		1		1	1		1														1			1	1	1	1	1		
Patterson 2013	1	1		1				1		1	1		1					1				1			1					1		1			1	
Pinelli 2015											1	1		1					1		1				1			1	1	1	1	1	1	1	1	

Journal of Health Services Research & Policy
The social networks of hospital staff: a realist synthesis
Claire Blacklock, Amy Darwin, Mike English, Jacob McKnight, Lisa Hinton, Elinor Harriss and Geoff Wong

Document	1	2a	2b	3	4	5a	5b	6a	6b	6c	7a	7b	7c	8a	8b	9a	9b	10a	10b	11a	11a	11b	11c	12a	12b	13	14	15	16	17	18a	18b	19a	19b	20a	20b
Rangachari 2008						1		1		1	1	1	1	1	1			1		1		1	1	1	1	1	1			1	1	1	1	1	1	1
Reed Elliot 2012								1		1	1	1						1							1						1	1	1	1	1	1
Regts 2016						1		1		1	1		1	1											1						1		1	1		
Riley 2009				1				1	1	1	1			1	1			1							1		1			1	1	1	1	1	1	1
Shafiei 2018	1	1	1	1		1	1	1	1	1			1	1		1				1		1		1	1					1	1	1				
Shoham 2015		1						1		1	1	1	1	1				1	1	1	1	1	1				1			1	1	1				
Shoham 2016	1	1		1							1	1		1	1	1		1	1	1		1								1	1		1	1		
Sreeramoju 2018						1		1		1	1		1																							
Skyes 2015	1			1		1	1	1	1	1	1	1	1	1	1		1	1							1		1	1	1	1	1	1	1	1	1	
Sykes 2011	1	1	1	1		1	1	1	1	1	1		1	1	1			1							1					1	1	1	1	1	1	1
Tasselli 2015	1	1	1	1	1	1		1	1	1	1	1	1	1	1	1	1	1	1	1	1	1	1	1	1	1	1	1	1	1	1	1	1	1	1	
Tsang 2012	1	1		1		1		1		1			1	1				1							1	1	1	1			1	1	1	1		
Verkatesh 2011	1	1		1	1	1	1	1	1	1	1		1	1			1		1					1	1		1				1	1	1	1	1	
Wagter 2012	1	1	1	1		1		1	1	1	1	1	1	1				1	1	1		1	1	1	1	1		1	1	1	1	1	1	1		
Walton 2010								1	1	1	1			1	1		1	1	1		1		1		1	1			1		1	1	1	1	1	1
West 1999	1	1	1	1	1	1	1	1	1	1	1		1	1	1	1	1	1		1	1	1	1	1	1		1			1	1	1	1	1		
West 2005	1	1	1	1	1			1		1				1	1	1	1	1		1		1	1	1	1		1		1	1	1	1	1	1	1	1
Westbrook 2007	1	1	1								1																			1						
Yi 2017	1	1			1			1	1	1				1	1					1					1	1				1	1	1	1	1	1	1
Yuce 2014	1				1									1	1			1			1		1													

Journal of Health Services Research & Policy
The social networks of hospital staff: a realist synthesis
Claire Blacklock, Amy Darwin, Mike English, Jacob McKnight, Lisa Hinton, Elinor Harriss and Geoff Wong

(E) Prisma diagram

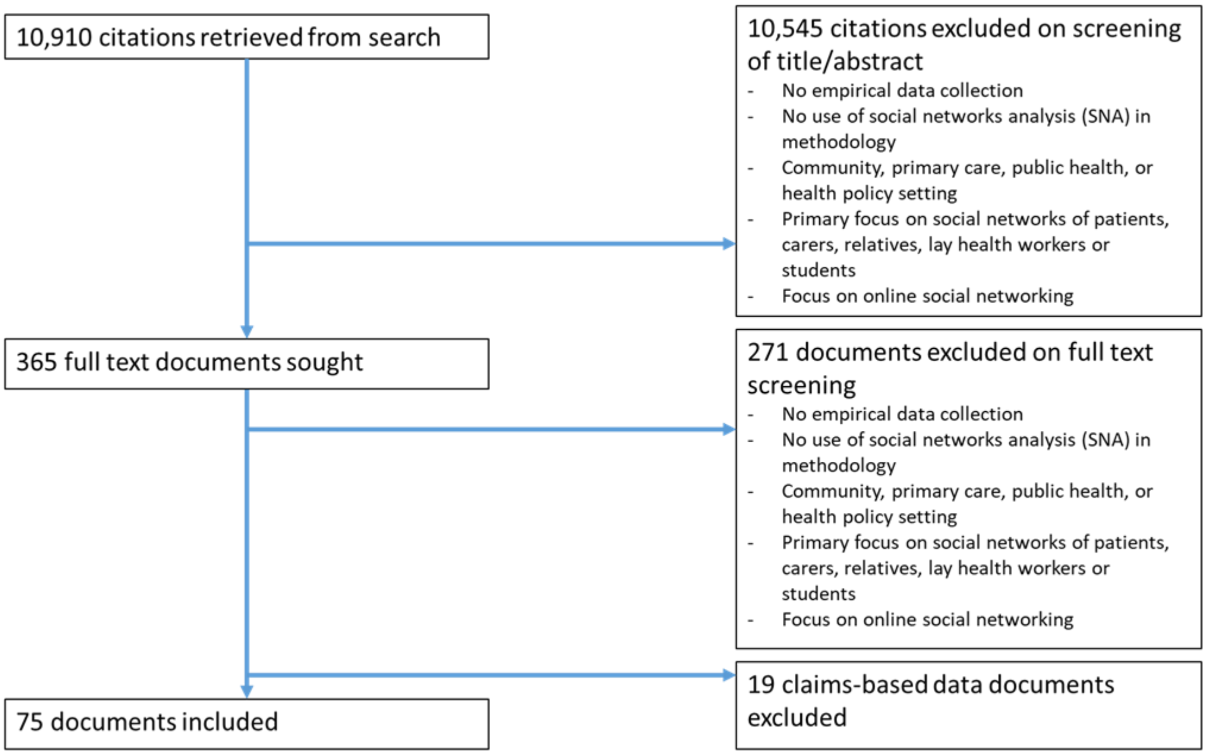

(F) Characteristics of included documents

	Author	Year	Country	Participants	Social network data collection	SNA measures	Main findings
1	Anderson[1]	1985	US	Physicians of a private group practice in a private, university affiliated, teaching hospital in US	Roster interview method identifying professional contact between individuals, by self-reported communication activity over the preceding 6 weeks. Demographic and computer usage data collected from hospital records.	Density<br>'Sender, Relayer, or Receiver' (based on ratio of in-degree vs out-degree)<br>Prestige (In-degree)	Four sub-groups of physicians were identified, each displaying similar communication structures, similar demographics and similar computer usage. Sub-groups formed an overall core-periphery pattern.
2	Anderson[2]	1991	US	Nurses working in surgical unit of private general hospital	Name generator method to identify colleagues on unit who most often provided them with support when under stress	Path distance matrices<br>Centrality<br>Distance from centre	Nurses under stress tend to look for support from nurses who are like themselves in some way, and are also experiencing stress or burnout. Nurses under stress were centrally located on the day shift, and more peripherally located on evening and night shifts. Nursing managers were not always centrally positioned in the network (varied by shift), impacting on their ability to provide support.
3	Anderson[3]	2011	US	Operating room staff of general surgical and neurosurgical specialities large teaching hospital in US	Staffing and case data from all operations in 2008	Core-periphery structure, Coreness<br>Centrality: Closeness, Betweeness, Eigenvector	Both specialties showed a core and periphery structure, more pronounced in neurosurgery. Core staff tended to work on longer cases, earlier in the day, and in larger teams. Nurses were disproportionately core staff.
4	Altalib[4]	2017	US	Neurologists and other health care providers to patients with epilepsy in US.	Patient-sharing data from electronic health records of patients with epilepsy/convulsions.	Node degree<br>Adjusted node degree	Connectivity increased over time, with networking within and between facilities.
5	Bae[5]<br>(Bae et al., 2017)	2017	US	Oncology nurses in an acute care hospital in the US	Roster method to explore patterns of assistance at work and demographic electronic survey.	Centrality: In-degree, Out-degree, In-closeness, Out-closeness, Betweenness, Eigenvector, Shortest path in, Shortest path out<br>Other: Density, Reciprocity, Transitivity	Help was perceived to be given more than received. Shift (day >night) and education level (less for degree holders) were significantly associated with amount of help received by each nurse. Nurses working overtime provided more help to colleagues.
6	Barrot[6]	2013	US	Anethesiologists in charge of purchasing equipment at a specific hospital, as identified as manufacturers contact person	Survey in which asked to name up to 6 people in work-related network, and the connections those people might have with one another	Centrality	Clustering of purchasing anesthesiologists<br>Network overall between purchasing anesthesiologists at different hospitals was fragmented, however there was one main component, and one central node. Some individual purchasing anesthesiologists were isolated
7	Benham-Hutchins[7]<br>)	2010	US	Staff involved in patient transfers (hand-offs) between departments in an acute care hospitals in the US.	Questionnaires completed by staff about communication activity between themselves and colleagues during five patient hand-offs.	Individual (provider): betweenness centrality, closeness centrality, eigenvector centrality and total degree centrality<br>Network (handoff): betweenness centralization and hierarchy measures	Information exchange during hand-offs were found to be nonlinear, unpredictable and shaped by the information needs of the providers. Patient handoffs often coincided with shift changes, adding further complexity to communication.

	Author	Year	Country	Participants	Social network data collection	SNA measures	Main findings
8	Boyer[8]	2010	France	Staff working in the psychiatry department of a French public hospital	Name generator method used to identify with whom individual staff have ties and with whom they would discuss important matters. UCINET software used in analysis	Centrality (Out-degree)<br>Prestige (In-degree)<br>Cliques (cluster analysis)	Centrality was associated with job title (physician vs. nurse/psychologist/administrator) and work activity (transverse working). Prestige and clique participation (no of cliques per actor) were associated with male gender, job title and work activity.
9	Brewer[9]	2018 (a) HER DJ	US	Staff in twenty four nursing units in three US acute care hospitals with different physical space design/layouts at four points in time over a 7-month period	Roster method used in questionnaire to identify communication patterns between nurses. Patient fall rates and style of physical space design/layout were also measured	Node size, density, weighted density, total degree centrality, betweenness centrality, eigenvector centrality, clustering coefficient, average distance, diffusion	Nursing unit design was associated with different communication patterns between nursing staff, and also with patient falls.
10	Brewer[10]	2018 (b) JNA	US	Staff in twenty four nursing units in three US acute care hospitals with different physical space design/layouts at four points in time over a 7-month period	Roster method used in questionnaire to identify communication patterns between nurses. Patient fall rates and style of physical space design/layout were also measured	Node size, density, weighted density, total degree centrality, betweenness centrality, eigenvector centrality, clustering coefficient, average distance, diffusion	Larger teams were associated with more medication errors and patient falls. More patient-related communication was associated with fewer medication errors and lower fall rates.
11	Cerne[11]	2004	Slovenia	Nurses working in four Slovenian hospitals	Ring method used to identify contacts in 1st, 2nd and 3rd social rings of closeness.	Closeness of rings	Nurses wanted a larger network. Nurses trusted those colleagues in the closest rings more, who were likely to be peers. They were more likely to discuss major decisions or emotional support with those in the closer ring. Trust and respect were linked to those in closer rings.
12	Chan[12]	2017	Australia	Geriatric wards in four tertiary referral urban teaching hospitals in Australia.	Roster based method was used in staff questionnaire to identify communication about medication advice. Medical records were assessed for drug errors.	Cohesiveness, density, degree centralisation, hierarchy value, transivity<br>Evaluation of importance based on multi-indicator (EIMI) method	Junior doctors, senior nurses and pharmacists acted as links. Junior doctors linked to senior doctors. Doctors were often sought for medication advice. Junior doctors often sought one another. Apart from the ward-based pharmacist, other allied health professionals were peripheral.
13	Cott[13]	1997	Canada	Healthcare workers on three ward teams at multilevel geriatric care facility in Canada; nursing home ward, chronic care hospital ward, and palliative care ward.	Roster method was used by questionnaire. Block modelling was performed in SPSS	Block modelling<br>Density within blocks	Senior nurses are linked to multidisciplinary professionals and are involved in decision-making work, wherease lower level nurses are involved in task-orientated work.
14	Creswick[14]	2009	Australia	Staff in the emergency department of a teaching hospital in Australia	Roster method was used to identify colleagues who were sought in problem-solving, medication advice-seeking and socialising. UCINET and NetDraw used	Connectedness<br>Isolates<br>Degree centrality (in-degree, out-degree)<br>Betweenness centrality<br>Degrees of separation<br>Reciprocity	The unit was highly connected overall (except socialising), with professionals however tending to be more connected to those in their own professional group. Senior nurses and doctors occupied central positions, particularly doctors in the medication prescribing network.

	Author	Year	Country	Participants	Social network data collection	SNA measures	Main findings
15	Creswick[15]	2010	Australia	Staff of renal ward of teaching hospital in Australia.	Roster method used in questionnaire, focusing on medication advice-seeking. UCINET and Netdraw software were used in analysis	Cohesiveness (geodesic distance)<br>Density<br>Tie strength<br>Reciprocity<br>In-degree, Out-degree, Degree centrality<br>Betweenness centrality	Relatively infrequent medication advice-seeing was seen, with professional group cliques. Reciprociity of advice-seeking was 30%. Hierarchy (not based on seniority) was seen, where key individuals were sought for advice, as well as gatekeepers.
16	Creswick[16]	2015	Australia	Staff of renal and respiratory ward of Australian teaching hospital.	Roster method – for every colleague respondents asked to indicate frequency of medication advice seeking from that colleague.	Density, reciprocity, in-degree, centrality ('hubs'), interprofessional communication density,	Medication advice-seeking was mostly directed to pharmacists, junior doctors and senior nurses, with senior doctors peripheral or even disconnected to the rest of the network.
17	Currie[17]	2014	UK	Hybrid mid-level managers, senior managers and professionals in three hospitals, and external producers of patient safety knowledge in the UK.	This was a mixed-methods study, comprising qualitative interviews, SNA and quality improvement activities.<br>Name generator used as part of semi-structured interviews. Snowballing used to identify further participants. UCINET software used in analysis	Centrality<br>Fernandez and Gould typology analysis of brokerage roles	Nurses found to occupy gatekeeping roles, with two doctors occupying a coordinating role in the overall management network.
18	Dauvrin[18]	2015	Belgium	Staff in five primary care services and four hospitals.	Roster method used to construct social networks. Competences in Ethnicity and Health questionnaire was used to measure cultural competency.	In-degree centrality used to identify leaders	Some elements of cultural competence of staff were linked to cultural competence of leaders. Leaders identified by social networks analysis were not always the formal leaders
19	Dauvrin[19]	2017	Belgium	Staff in five primary care services and four hospitals, from geriatric, intensive care, oncology, psychiatric, communicable diseases, palliative care and endocrinology units.	Self-administered questionnaire divided into three parts: cultural competence, including three distinct dimensions, social relationships and sociodemographic data.	Reciprocity<br>Popularity (in-degree centrality)<br>Socialness (out-degree centrality)	Cultural competence did not seem to be a socially shared phenomenon, however awareness of exposure to cultural situations was.
20	Di Vincenzo[20]	2017	Italy	Physicians in 6 hospitals of a Local Health Authority in Italy	Name generator method used in paper questionnaire. UCINET software used. Number of publications per doctor also collected as part of assessment of knowledge level and ego-network redundancy.	Network constraint	Doctors tended to seek advice from those with greater knowledge prestige than them, and in same speciality. This was associated with concept of redundant networks. Effect moderated by being in the same professional group, e.g. manager
21	Effken[21]	2013	US	Nursing staff from seven medical-surgical patient care units from three acute care hospitals in the US, involved in shift handovers.	Roster method used to identify colleagues with whom an individual gave and received information with at handover. Patient safety outcomes were also measures. *ORA used in analysis	Clustering coefficient<br>Component count: strong<br>Component count: weak<br>Density<br>Diffusion<br>Fragmentation<br>Hierarchy<br>Isolates<br>Centrality in-degree<br>Eigenvector centrality<br>Cliques	Stronger connections among handoff staff were associated with fewer falls. Similarly, more clusters or small groups, cliques and triads were associated with fewer adverse drug events. Different associations were seen in regards to patient satisfaction and symptoms.

	Author	Year	Country	Participants	Social network data collection	SNA measures	Main findings
						Triads	
22	Espinoza[22]	2018	Chile	Healthcare providers at a large hospital in Chile, who had worked with the same clinical team for a minimum of 6 months.	Mixed methods study including surveys relating to satisfaction and team climate, interviews and social network analysis. UCINET software was used in analysis.	Density<br>Isolates<br>Centrality<br>Sub-groups	Team climate predictor of team satisfaction, as well as length of time working with team. Highly satisfied teams were more closely connected and centralised than least satisfied. Nurse leaders, common goals, focus on patient-centred care, communication style, fostering innovation and focus on task with clear team roles were all found to be important.
23	Fong[23]	2017	US	Three intensive care units in a teaching hospital in the US	Name generator self-administered social network survey relating to trust, advice seeking, support, help, confiding and influence ties.	k-Means clustering to identify influencers	Three typologies of influencers found: 'well-rounded', 'relational' and 'knowledge- or skill-based' influencers, which have characteristics of particular relevance to an ICU.
24	Forsyth[24]	2018	US	Pre-operative team briefings in the surgical gynaecology department of a quaternary care hospital in the US	Video-recorded team briefings were used to measure communication flow between team members. Data analysed using Code XL	Graph density<br>Reciprocated edge ratio<br>In-degree centrality, out- degree centrality	Surgeon-led team briefings were most common, with communication from surgeon dominant and little communication between other members. Resident-led briefings were still dominated by surgeon, however other team members communicated more. Anaesthetists were the least involved in briefings (sometimes not even present)
25	Grace[25]	2015	US	Staff of neonatal intensive care unit of a children's teaching hospital in the US.	Roster method used in face-to-face interview to identify "highly productive working relationships". Measures of distress and burnout and social support also collected.	In-degree<br>Reciprocity	Both network position and perception of social support are important (and not interchangeable) for reducing workplace distress. Perception of social support is also important for reducing burnout
26	Heiligers[26]	2008	Netherlands	Part-time and full-time doctors working in teams in internal medicine, surgery and radiology in the Netherlands	Postal questionnaire with three question domains: consulting, communication or (intended) relationships of trust with partners within their own team, and perceived socio-emotional relationships. Semi-structured interviews were also conducted with a representative from each team on individual and team characteristics	Ego-network size<br>Reach efficiency	Size of network and trust did not differ between full-time and part-time doctors. Reach-efficacy was lower in part-timers. Communication was higher in full-time teams. There was no differences in consulting networks. Team size, formal leader and age were associated with communication network size and trust relationships.
27	Heng[27]	2005	Australia	Department heads in a tertiary referral hospital in Australia, with a focus on facilities management.	Name generator used in questionnaire.	Density, degree centrality, betweenness centrality, structural holes measure	Facilities managers occupy central positions in hospital facilities networks. They occupy structural holes and connect across disciplines. Their work is central to the functioning of the hospital.
28	Heng[28]	2013	Australia	Snowball sample from facilities management group in a tertiary referral hospital in Australia	Name generator to identify key contacts in other departments with whom aspects of facilities management are discussed. Strength of tie and content/purpose of	Density, degree centrality, betweenness centrality<br>Brokerage potential across structural holes: effective size, efficiency,	Facilities management departments were seen to broker information between different departments of the hospital and to connect otherwise disconnected areas. A few facilities management departments were found to be particularly powerful in their

	Author	Year	Country	Participants	Social network data collection	SNA measures	Main findings
					communication also collected. UCINET software used in analysis. Semi-structured interviews also conducted.	constraint and hole signature.	network position in the hospital.
29	Hurtado[29]	2018	US	Registered nurses and certified nursing assistants from the medical/surgical and intensive care units of a community hospital in the US	Roster method social networks survey administered by research assistants to identify peer support for safe patient handling, as well as data on usage of manual handling equipment.	In-degree, out-degree, reciprocation	Use of equipment was associated with out-degree and reciprocal nominations. In-degree nominations were not associated with use of equipment.
30	Iacopino[30]	2018	Italy	Physicians and nurses in three surgical wards in Italian NHS	Questionnaire in which respondents asked to name colleagues or other professionals with whom they exchanged advice about a new surgical system.	Dyadic approach to analysis. Same adoption, connectedness, ego network size similarity, ego network composition similarity, ego network constraint similarity.	Similar perceptions relating to technology were associated with social connections, particularly those ties displaying homophily and similar network constraint.
31	Iyengar[31]	2011	US	Physicians prescribing a new drug associated with potential high risk in three large cities in the US	Name generator method used as part of a postal survey to identify discussion and referral ties (conducted by a drug company). In addition prescribing data collected for 17 months after the new drug was introduced.	Visualisation of sociogram	When controlled for sales calls and individual characteristics of the physician, earlier adoption was associated with network and self-reported leadership. Physicians with lower leadership scores were found to be more influenced by peers. More central physicians and those who prescribed more (and therefore more credible), were found to be the most influential in the network. Sub-groups were seen of physicians of different ethnic backgrounds, with a single broker between the two groups.
32	Iyengar[32]	2015	US	Physicians prescribing a new drug associated with potential high risk in three large cities in the US	Name generator method used as part of a postal survey to identify discussion and referral ties (conducted by a drug company). In addition prescribing data collected for 17 months after the new drug was introduced.	In-degree centrality	Discussion and referral ties found to influence the trial of a new drug by physicians (particularly those with low self-reported leadership), but not repeat prescribing of that drug. Close colleagues (i.e. working in the same institution) have a peer influence on prescribing, moderated by status, which increases with time.
33	Janmano[33]	2018	Thailand	Staff of a district hospital in Thailand, which included male and female general medical wards, an intensive-care unit (ICU), and a private medical ward.	Name generator as part of interview on medication advice-seeking. UCINET software used in analysis. Data on medication error reporting also collected.	In-degree, out-degree, Betweenness centrality	Similar out-degree across cadres. Pharmacists and physicians were most central in network (in-degree). However different centralities for different aspects of medication chain. Females and those with lower degrees were more likely to report errors. In-degree centrality was the most influential characteristic for reporting medication errors in the hospital inpatient medication system
34	Jippes[34]	2010	Netherlands	Supervisors and residents in obstetrics–gynecology and paediatrics in the Netherlands	Social networks analysis conducted of supervisors with their fellow medical specialists in their own departments, using web-based questionnaire. Medical specialists rated their communication intensity.	Degree centrality, betweenness centrality, closeness centrality	Social ties were more important than formal training in adoption of new innovation (giving feedback in postgraduate clinical training). Strength of tie, both strong and weak ties and age were important.
35	Jippes[35]	2013	Netherlands	Supervisors and residents in four clinical training programmes in the Netherlands: obstetrics–gynecology, paediatrics,	Social networks analysis conducted of supervisors, using web-based questionnaire. In addition, data were collected on provision of feedback, attendance at 2-day teach-the-teacher course by supervisors, as well as on	Centrality	The clinical supervisor's centrality within his or her department's social network was significantly related to both self-assessed and resident-assessed innovation adoption scores, whereas attendance at the 2-day training course was only related to self-assessed adoption.

	Author	Year	Country	Participants	Social network data collection	SNA measures	Main findings
				radiology, and anesthesiology.	feedback received by residents.		
36	Landim[36]	2010	Brazil	Nurses working in a haematology unit in Brazil.	Name generator method used (only 10 staff included, but 25 more named). UCINET software used in analysis.	Density, reciprocity, centrality	Group density found to be very low, suggesting difficulty in group working and processes of achieving consensus, as well as limited support on problem-solving.
37	Lindberg[37]	2013	US	Hospital-based outpatient haemodialysis unit in the US.	A social networks survey was conducted before and after the intervention, in addition to other methods of process and outcome data collection. Intervention was a quality improvement collaborative established, involving 21 haemodialysis facilities. This intervention included “positive deviance” team quality-improvement activities to reduce hospital-acquired infections.	Connectivity, inclusion, reach, centralisation	Following the intervention, staff social networks were found to be larger, and the network for collaborative ties more centralised.
38	Llupia[38]	2016	Spain	Healthcare workers from two hospital departments following an influenza vaccination campaign at a hospital in Spain	Name generator method during face-to-face interviews focusing on who individuals talked to or shared activities with during the vaccination campaign.	Dyadic analysis: sender, receiver, mutuality	Links were more likely between healthcare workers from the same profession, sex, age, and department, but not between healthcare workers who shared the same vaccination behaviour. Older and vaccinated HCWs were more likely to be named by interviewees.
39	Lurie[39]	2009	US	Staff working in intensive care unit in hospital in the US.	Observation of team rounds, followed by a survey using roster method to identify frequency of communication with colleagues and patients’ family members.	Visual inspection of sociograms	Two different teams were observed for one unstable and one stable patient. The staff networks around the two patients were observed to be very different, with a physician being central in the first team, which is denser with the family more connected (unstable patient), and nursing staff central in the second, which is less dense with the family more peripheral
40	MacPhee[40]	2000	US	Nurses working flexible and traditional schedules in a tertiary referral paediatric hospital in the US.	Name generator with closeness rings diagram used as part of a survey. Emotional support and satisfaction variables also measured.	Position of alter in social ring structure	Flexible and traditional nurses were found to report similar social networks. Colleagues who were closest and provided the most support to nurses were peers, rather than managers.
41	MacPhee[41]	2002	US	Nurses employed in ten rural hospitals and one urban hospital in the US.	Name generator and social network circle diagram used in questionnaire, ranking and rating named colleagues for nine types of personal support.	Position of alter in social ring structure	Three levels of nurse managers were identified, with each level providing specific types of support to (and from) nurses. Nurses generally felt well supported.
42	Marquez-Sanches[42]	2018	Spain	Healthcare employees at a public hospital (Surgical Unit, Dialysis Unit, Management Team, General Medicine, Microbiology Laboratory, Paediatric Unit and Intensive Care) and a primary healthcare centre in Spain	Name generator and roster method used to identify colleagues with whom individuals sought advice on career and working environment. UCINET software was used in analysis. Team performance ratings were also collected.	Out-degree ties (Split into those within the organisation - internal, and those outside of the organisation - external)	External ties were found to improve the performance of physicians at both the individual and team level. In contrast, external ties did not improve work performance of nurses.

	Author	Year	Country	Participants	Social network data collection	SNA measures	Main findings
43	Mascia[43]	2011 (a) BMC	Italy	Physicians employed in six hospitals in an Italian local health authority (LHA).	Name generator used in questionnaire, including tie strength for advice ties. UCINET software was used. Attitudes towards evidence-based medicine were also measured.	Physician dyads	Homophily was seen to result in inter-physician collaboration, particularly the same field of specialization, co-authorship, affiliation with the same organizations, and similarities in attitude toward evidence-based medicine.
44	Mascia[44]	2011 (b) SSM	Italy	Physicians working across six hospitals in Italy	Name generator (with roster list for reference) used to identify colleagues with whom an individual discussed professional matters. Propensity to adopt evidence-based medicine was also measured.	Ego-network constraint indicator (controls: size, number of ties in ego-network)	Physicians who had highly constrained interpersonal networks were less likely to report adopting evidence-based medicine. The presence of structural holes in networks were associated with a greater likelihood of a physician using evidence-based medicine.
45	Mascia[45]	2013	Italy	Physicians employed in six hospitals in an Italian local health authority (LHA).	Name generator used in questionnaire, including tie strength for advice ties. UCINET software was used. Attitudes towards evidence-based medicine were also measured.	Network Coreness score, derived from centrality and interconnectedness	Those with less self-reported use of evidence-based medicine were more central in the network than those using evidence-based medicine.
46	Mascia[46]	2015	Italy	Physicians working across six hospital sites in an Italian local health authority (LHA).	Study used data from Mascia 2011(a), 2011(b) and 2013	Multiple regression-quadratic assignment procedure (MR-QAP), using physician dyads	Professional (speciality) homophily was found to be of stronger influence than institutional homophily. Doctors graduating less recently were seen to show fewer collaborative ties. Doctors holding managerial roles did not collaborate with others holding similar roles
47	McCurdie[47]	2018	Australia	Staff working in the intensive care unit of a hospital in Australia.	Participant observation of 'primary participants' for a minimum of 3 hrs, looking for intersections/interruptions ('secondary participants'). Two-perspective method used to examine each interruption. Social network of interruptions constructed from data using Gephi software.	In-degree, out-degree, standardised in-degree, standardised out-degree, weighted in-degree, weighted out-degree, sociometric status, generalised in-degree centrality, generalised out-degree centrality, network density, modularity	The in-charge nurse role was the most frequently interrupted. However the bedside nurse role initiated most interruptions, and interrupted every other role in the ICU. Team-leader and in-charge roles had high in-degree values, and had sole access to keys for the restricted medications cupboard, which the bedside nurse was unable to access without. Nursing roles were highly connected.
48	Meltzer[48]	2010	US	Physicians working in the general inpatient medicine services at a hospital in the US.	Roster method to identify frequency and quality of interactions with physician colleagues.	Degree centrality, net degree, density, betweenness centrality	Sub-groups of physicians by speciality, gender, self-reported hospitalist status, and research activity were seen. More connected physicians communicated with other more highly connected physicians. Non-redundant connections held by a specific group is explored.
49	Menchick[49]	2010	US	Physicians working at 6 academic medical centres of different sizes in the US	Medication advice-seeking networks constructed for high-esteem and low-esteem hospitals. Structurally equivalent positions were compared between high and low esteem hospitals.	Esteem: In-degree centrality, adjusted by number of physicians in hospital (population size)	Within high-esteemed hospitals, physicians from higher esteemed medical schools, and those doing less clinical work were held in higher esteem. In lower-esteemed hospitals, physicians who read across journals and engaged in more patient care were more highly esteemed. Stigma was associated with graduating from a high-esteemed medical school but now working in a low-esteemed hospital.
50	Myers[50]	2016	US	Surgical teams in a large academic teaching hospital	Dataset of surgical procedures, sharps-related percutaneous blood and body fluid exposures (BBFE) and index of collaboration, based on social network measures applied to surgical team data.	Team stability index score	Incidence of BBFE reduced with greater team collaboration score. Not much absolute reduction in real terms, but was significant for non-suture needle device related BBFE (and large numbers in analysis)

	Author	Year	Country	Participants	Social network data collection	SNA measures	Main findings
51	Pappas[51]	2003	US	Middle managers in a hospital in the US	Roster method of measuring communication network of mid-level managers. Measures of strategic consensus, based upon qualitative interviews with management team, and measures of managers' knowledge of the internal and external organisational environments were also collected.	Degree centrality	Knowledge of the internal workings and vision of the organisation is important for consensus building, as well as the social standing of the manager in the organisation
52	Pappas[52]	2004	US	Middle managers at a hospital in the US.	Qualitative interviews, followed by a survey. Roster method used in survey to identify communication and friendship networks. Middle manager activities and strategic knowledge were also measured.	Centrality	Information flow was found to be controlled by those managers with close relationships. Social position in informal networks was found to mediate the association between 'strategic knowledge' and 'upward-orientated influence' of middle managers.
53	Pappas[53]	2007	US	Middle managers in a hospital in the US	Roster method of measuring communication network of mid-level managers. Measures of strategic activity, based upon qualitative interviews with management team, and measures of role boundary-spanning activity were also collected.	Degree centrality, closeness centrality, eigenvector centrality	Divergent strategic behaviour is undertaken more by those who are more central in the social structure, and also by those who are boundary spanners (though not exclusively). Socialisation with diverse people allows one to 'learn' innovation.
54	Patterson[54]	2013	US	Emergency department staff in a hospital in the US.	Roster method using a survey was used to construction social networks of communication about problem-solving, medication advice, and socialising.	Network density<br>Network centralisation<br>In-degree centrality	Variation was seen in communication over time, shift, and for different purposes. General problem solving communication was also associated with medication advice-seeking and socialising (however these two were only loosely linked themselves).
55	Pinelli[55]	2015	US	Patient discharges from a medical unit at a university teaching hospital in the US.	Electronic medical records for seven patient discharges were used and providers were asked with whom they communicated by chain interview technique.	Density, betweenness centrality	Junior doctors were most central in the discharge process, which was a multidisciplinary event.
56	Rangachari[56]	2008	US	Purposive sample of two good-performing hospitals and two poor performing hospitals in the US. Performance was assessed specifically in regards to medical coding.	Roster method using a survey was used to construct knowledge sharing network in regards to quality measurement and hospital coding performance.	Structural equivalence analysis (SEA) and visualisation of SEA	A stark difference was seen in communication structure between good performing and poorer performing hospitals. In good performing hospitals there was better interdisciplinary communication, with senior managers taking a proactive central role, and measures taken to raise the status of medical coding in the hospital.
57	Reed Elliot[57]	2012	Brazil	Staff in four hospitals in Brazil	Roster method used to identify ties and tie strength. Block modelling was used in the analysis.	Concor groups/"blocks"	Formal hierarchies did not predict block modelling findings. Hospitals varied as to who was in each block/core. Core-periphery patterns were seen. This is attributed by the authors to the history of the organisations and related to institutional culture.
58	Regts[58]	2016	Netherlands	Medical and orthopaedic nurses in four hospitals in the Netherlands.	Roster method to identify advice network of nurses Personality traits, job satisfaction and performance also measured.	Affect-based network centrality (combination of in-degree and out-degree centrality)	Study found an interaction between certain personality traits (neuroticism and introversion and stable extraversion) and benefit from network position for satisfaction and performance.

	Author	Year	Country	Participants	Social network data collection	SNA measures	Main findings
59	Riley[59]	2009	Australia	Operating room departments in three hospitals in Australia	Ethnographic study comprising observations and qualitative interviews, examining the interactions between operating room staff, to identify gatekeeping roles.	Ethnographic data	Gatekeeping activities of theatre nurses explored, and findings grouped into: Protecting; Facilitating and Disseminating; Providing Access; and Negotiating Social/Cultural Position. Associations between gatekeeping and power discussed, as well as the potential impact of gatekeeping activities on patient care.
60	Shafiai[60]	2018	Iran	Nurses from six departments in a hospital in Iran	Data for construction of social networks gathered from qualitative interviews with nurses.	Degree centrality, closeness centrality, betweenness centrality, eigenvector centrality	Nurses formed cliques within their own departments. Different departments had different communication structures/density, with surgery the most dense. There was little communication across departments, with just a few central nurses
61	Shoham[61]	2015	US	Staff working in a burns unit in the US	Survey administered to staff to elicit frequency of discussing patient care with colleagues in a 24 hour period.	Out-degree centrality, in-degree centrality, betweenness centrality, density	Junior doctors were found to occupy central positions, despite frequent rotations. Staff communication was found to cluster around two groups of patients (ill vs less ill).
62	Shoham[62]	2016	US	Staff working in a burns unit in the US	Survey administered to staff to elicit frequency of discussing patient care with colleagues in a 24 hour period.	Degree centrality, dyadic ties	The burns unit was generally well connected across different professional groups. However, Nurses tended to communicate more with other nurses. Doctors tended to be more central overall.
63	Sreeramoju[63]	2018	US	Healthcare staff on six medical wards at an academic hospital in the US.	Intervention study to improve patient safety culture related to infection prevention. Three wards were assigned to a social change intervention, implemented over nine months.<br>A social network survey was conducted at 6, 15 and 24 months, to identify which colleagues an individual collaborated with to prevent infections, colleagues with whom they were involved in projects, colleagues who inspire them, and colleagues with whom they would like to work in the future. Patient safety culture and rates of hospital-acquired infection were also measured.	Degree centrality	Core staff in preventing hospital infections were identified as the patient's nurse, patient care technician, charge nurse, ward manager, and ward clerk. There was no deterioration in hospital-acquired infections in intervention arm.
64	Sykes[64]	2011	US	Doctors working in a private hospital in the US	Roster method used in survey to identify advice networks. Adoption of electronic medical records was also measured.	Degree centrality, eigenvector centrality (adapted)	The more central doctors were more likely to be associated with higher patient satisfaction, but were less likely to adopt electronic medical records. This was hypothesised by authors to be due to ease of alternative communication pathways and compliance with organisational norms.
65	Sykes[65]	2015	Australia	Surgical procedures involving staff members across four surgical teams (general, thoracic, orthopaedic, and paediatric) in a large tertiary hospital in Australia	Data were retrospectively extracted using the electronic database Operating Room Management Information System (ORMIS). Gephi software was used in analysis.	Edges between nodes to create a visual map only	A small number of staff were shared across the teams. Longer procedures involved more staff, some of whom rotated during the procedure itself
66	Taselli[66]	2015	Italy	Doctors and nurses of the nephrology department of a hospital in Italy	Roster method was used to identify professional knowledge exchange networks. Qualitative interviews were also conducted	Average degree centrality, hierarchy score, average betweenness centrality, closeness centrality, brokerage	Structure of professional networks of nurses and doctors found to be very different, with strong professional boundaries. The roles of mediators and brokers in knowledge exchange explored.

	Author	Year	Country	Participants	Social network data collection	SNA measures	Main findings
					to explore aspects of knowledge transfer		
67	Tsang[67]	2012	Taiwan	Nurses in dialysis department of a medical centre in Taiwan	Social networks data relating to information exchange at work and sharing of personal issues. Organisational citizenship behaviour, workplace stress and work satisfaction were also measured.	Degree centrality	Work network centrality positioning was different to friendship network centrality. Centrality was associated with organisational citizenship behaviour, which also reduced work stress and increased work satisfaction. Expected reciprocity associated with organisational citizenship behaviour was discussed by authors.
68	Venkatesh[68])	2011	US	Staff working in a hospital in the US, after the implementation of a new IT system	Roster method used as part of a staff questionnaire, to identify advice networks. UCINET software used in analysis. Data on IT system use and patient satisfaction also collected.	In-group degree centrality<br>Out-group degree centrality	A different influence for centrality on IT system use was found in different professional groups. Ties among doctors had a negative association with IT use, as did ties with doctors on IT system use by other staff. This is despite the use of the IT system being associated with better patient satisfaction.
69	Wagter[69]	2012	Netherlands	Staff of intensive and medium care unit in a large teaching hospital in the Netherlands	Roster method used to identify inter-professional learning networks.	Density, reciprocity, tie strength	The underlying network structure was found to consist of two distinct networks, with a single group of nurses being isolated. Staff were found to learn from within their own profession, particularly from senior doctors. Authors comment that opportunity (e.g. physical proximity) and homophily contributed to explain the patterns seen.
70	Walton[70]	2010	Canada	Staff involved in ward rounds on general paediatric wards of a tertiary care, university-affiliated children's hospital in Canada	Non-participant observation of eighteen ward rounds (noting all verbal interactions). Self-administered survey also collected ratings of the primary purpose of ward round, its utility for patient care, education and team administration, and the degree of disruption caused by the presence of the observer	Freeman normalised graph centrality index	Ward rounds conformed to patterns, and were often dominated by the same individuals. Time taken per patient was longer at the start than at the end of the ward round. Perceptions of educational value by ward round attendees was higher than the authors' opinions.
71	West[71]	1999	UK	Clinical Directors of Medicine Directors of Nursing working in hospitals in England	Name generator used to identify people with whom the individual had spoken to about important professional matters, and how those identified were connected to one another.	Group degree centrality, actor information centrality	Directors of nursing were found to be in less dense networks, more hierarchical, with greater bridging/brokering role. Whereas medical directors were found to have denser networks which were less hierarchical, and were not seen to broker across structural holes in the same way as directors of nursing.
72	West[72]	2005	UK	Clinical Directors of Medicine Directors of Nursing working in hospitals in England	Name generator used to identify people with whom the individual had spoken to about important professional matters, and how those identified were connected to one another.	Category of alters	There were few doctors in the nurse managers' networks, and few non-doctors in the medical directors' networks. Both networks did however include some managers. Gender was found to be strongly associated with communication patterns, particularly amongst male doctors, who spoke to mostly other male doctors (also those similar in age and rank), but also as many male nurses as female nurses.
73	Westbrook[73]	2007	Australia	Staff working on three wards in a public hospital in Australia.	Roster method used to identify which colleagues individuals sought medication advice from, or socialised with.	Sociograms	Staff interacted mostly with those from the same professional groups, however some professionals were seen to occupy bridging roles, such as the pharmacist, junior doctors and ward clerk
74	Yi[74]	2017	China	Doctors working in outpatient department of a large children's hospital in China, which had recently implemented a new EMR system.	Roster method used to identify advice-seeking and advice-giving networks in regards to IT-related information. Data were also collected on electronic medical records usage.	Seeking-network closure<br>Giving-network closure<br>(Controls: seeking-network size, giving-network size)	Contacts who were connected with each other in advice-seeking networks, but not in advice-giving networks, were more likely to use electronic medical records.

	Author	Year	Country	Participants	Social network data collection	SNA measures	Main findings
75	Yuce[75]	2014	Turkey	Doctors employed across three divisions in state university hospital in Turkey	Free recall method used to construct advice-seeking networks for technological/computer-related and also clinical/professional matters.	In-degree centrality and Cattel's method to identify influential physicians	Medical advice-seeking networks were more cohesive than technical advice-seeking. Residents tended to go to mentors for advice, which may have presented a limit to IT adoption. Women were found to seek advice from women and men to seek advice from men.

References

1. Altalib, H. H., Fenton, B. T., Cheung, K. H., et al.. Care coordination in epilepsy: Measuring neurologists' connectivity using social network analysis. *Epilepsy Behav* 2017;*73*:31–35.

2. Anderson, C., Talsma, A. Characterizing the structure of operating room staffing using Social Network Analysis. *Nurs Res* 2011;*60*(6):378–385.

3. Anderson, J. G. Stress and burnout among nurses: A social network approach. *Soc Behav Pers* 1991;*6*(7):251–272.

4. Anderson, J. G., Jay, S. J. Physician Utilization of Computers: A Network Analysis of the Diffusion Process. *JOrgan Behav Manage* 1985;*6*:21.

5. Bae, S. H., Farasat, A., Nikolaev, A., et al. Nursing teams: behind the charts. *J Nurs Manag* 2017;*25*(5):354–365.

6. Barrot, C., Kuhlmann, J. A. N., Popa, AInfluence of Personal Communication Networks on Innovation Adoption – Using Multi-Agent Simulations to Project the Launch of an Innovative Medical Device. *Int J Innov Technol Manag* 2013;*10*(5):1.

7. Benham-Hutchins, M. M., Effken, J. A. Multi-professional patterns and methods of communication during patient handoffs. *Int J of Med Inform* 2010;*79*(4):252–267.

8. Boyer, L., Belzeaux, R., Maurel, O., et al. A social network analysis of healthcare professional relationships in a French hospital. *Int J Health Care Qual Assur* 2010;*23*(5):460–469.

9. Brewer, B B, Carley, K. M., Benham-Hutchins, M., et al. Nursing Unit Design, Nursing Staff Communication Networks, and Patient Falls: Are They Related? *HERD* 2018;*11*(4):82–94.

10. Brewer, Barbara B., Carley, K. M., et al. Relationship of Staff Information Sharing and Advice Networks to Patient Safety Outcomes. *J Nurs Admin* 2018;*48*(9):437–444.

11. Cerne, M. P. (2004). Social networks of hospital employees in nursing care. *Obzornik Zdravstvene Nege*, 2004;*38*(1):67–72.

12. Chan, B., Reeve, E., Matthews, S., et al. Medicine information exchange networks among healthcare professionals and prescribing in geriatric medicine wards. *Br J Clinl Pharmacol* 2017;*83*(6):1185–1196. https://doi.org/10.1111/bcp.13222

13. Cott, C. "We decide, you carry it out": a social network analysis of multidisciplinary long-term care teams. *Soc Sci Med* 1997;*45*(9):1411–1421.

14. Creswick, N, & Westbrook, J. I. Social network analysis of medication advice-seeking interactions among staff in an Australian hospital. *Int J Med Inform* 2010;*79*(6):e116-25.

15. Creswick, N, Westbrook, J. I., & Braithwaite, J. Understanding communication networks in the emergency department. *BMC Health Serv Res* 2009;*9*:247.

16. Creswick, N, Westbrook, J. I. Who Do Hospital Physicians and Nurses Go to for Advice About Medications? A Social Network Analysis and Examination of Prescribing Error Rates. *J Patient Saf* 2015;*11*(3):152–159.

17. Currie, G., Burgess, N., White, L., et al. A qualitative study of the knowledge-brokering role of middle-level managers in service innovation: managing the translation gap in patient safety for older persons' care. *HS&DR* 2014;*2*(32):1–118.

18. Dauvrin, M, Lorant, V. Cultural competence and social relationships: a social network analysis. *Int Nurs Rev* 2017;*64*(2):195–204.

19. Dauvrin, M, Lorant, V. Leadership and Cultural Competence of Healthcare Professionals. *Nurs Res* 2015;*64*(3):200–210.

20. Di Vincenzo, F., Mascia, D. Knowledge development and advice networks in professional organizations. *Knowl Manag Res Pract* 2017;*15*(2):201–213.

21. Effken, J. A., Gephart, S. M., Brewer, B. B., et al. Using *ORA, a network analysis tool, to assess the relationship of handoffs to quality and safety outcomes. *CIN* 2013;*31*(1):36–44.

22. Espinoza, P., Peduzzi, M., Agreli, H., et al. Interprofessional team member's satisfaction: a mixed methods study of a Chilean hospital. *Hum Res Health*, 2018;*16*:30.

23. Fong, A., Clark, L., Cheng, T., et al. Identifying influential individuals on intensive care units: using cluster analysis to explore culture. *J Nurs Manag* 2017;*25*(5):384–391.

24. Forsyth, K. L., Hildebrand, E. A., Hallbeck, M. S., et al. Characteristics of team briefings in gynecological surgery. *Appl Ergon* 2019;78:263-269

25. Grace, M. K., VanHeuvelen, J. S. Ties Received, Support Perceived: A Test of the Theorized Relationships among Workplace Networks, Social Support, and Mental Health in a Neonatal Intensive Care Unit (NICU). *Soc Ment Health* 2015;*5*(2):106–127.

26. Heiligers, P. J. M., De Jong, J. D., Groenewegen, P. P., et al. Is networking different with doctors working part-time? Differences in social networks of part-time and full-time doctors. *BMC Health Serv Res* 2008;8:204

27. Heng, H. K., McGeorge, W. D., & Loosemore, M. Beyond strategy: exploring the brokerage role of facilities manager in hospitals. *J Health Organ Manag* 2005;*19*(1):16–31.

28. Heng, H. K. S., Loosemore, M. Structural holes in hospital organisations Facilities managers as intrapreneurial brokers in the tertiary health sector. *Eng Const Arch Manag* 2013;*20*(5):474–487.

29. Hurtado, D. A., Dumet, L. M., Greenspan, S. A., et al. Social Network Analysis of peer-specific safety support and ergonomic behaviors: An application to safe patient handling. *Appl Ergon* 2018;*68*:132–137.

30. Iacopino, V., Mascia, D., & Cicchetti, A. Professional networks and the alignment of individual perceptions about medical innovation. *Health Care Manag Rev* 2018;*43*(2), 92–103.

31. Iyengar, R., Bulte, C. Van Den, Eichert, J., et al. How Social Networks and Opinion Leaders Affect the Adoption of New Products. *GfK Marketing Intelligence Review*, 2011;*3*(1):16–26.

32. Iyengar, R., Van den Bulte, C., Lee, J. Y. Social Contagion in New Product Trial and Repeat. *Marketing Science* 2015;*34*(3):408–429.

33. Janmano, P., Chaichanawirote, U., & Kongkaew, C. Analysis of medication consultation networks and reporting medication errors: a mixed methods study. *BMC Health Serv Res* 2018;*18*(1):221.

34. Jippes, E, Achterkamp, M. C., Brand, P. L. P., et al. Disseminating educational innovations in health care practice: Training versus social networks. *Soc Sci Med* 2010;*70*(10):1509–1517.

35. Jippes, E, Steinert, Y., Pols, J., et al. How do social networks and faculty development courses affect clinical supervisors' adoption of a medical education innovation? an exploratory study. *Acad Med* 2013;*88*(3):398–404.

36. Landim, F. L. P., Fernandes, A. M., Mesquita, R. B. de, et al. Interpersonal Network Analysis: Application to the Reality of a Nursing Team Working in a Hematology Unit. *Saude e Sociedade*, 2010;*19*(4):828–837.

37. Lindberg, C., Downham, G., Buscell, P., et al. Embracing collaboration: a novel strategy for reducing bloodstream infections in outpatient hemodialysis centers. *Am J Infect Controll* 2013;*41*(6):513–519.

38. Llupia, A., Puig, J., Mena, G., et al. The social network around influenza vaccination in health care workers: a cross-sectional study. *Implement Sci* 2016 ;*11* :7.

39. Lurie, S., Fogg, T., Dozier, A. Social Network Analysis as a Method of Assessing Institutional Culture: Three Case Studies, Acad Med 2009;84(8):1029-35

40. MacPhee, M. Hospital networking. Comparing the work of nurses with flexible and traditional schedules. *J Nurs Admin* 2000;*30*(4):190–198.

41. MacPhee, M, Scott, J. The role of social support networks for rural hospital nurses: supporting and sustaining the rural nursing work force. *J Nurs Admin* 2002;*32*(5):264–272.

42. Marques-Sanchez, P., Munoz-Doyague, M. F., Martinez, Y. V, et al. The Importance of External Contacts in Job Performance: A Study in Healthcare Organizations Using Social Network Analysis. *Int J Environ Res Public Health* 2018;*15*(7):27.

43. Mascia, D., Cicchetti, A. Physician social capital and the reported adoption of evidence-based medicine: exploring the role of structural holes. *Soc Sci Med* 2011;*72*(5):798–805.

44. Mascia, D., Cicchetti, A., Damiani, G. "Us and them": a social network analysis of physicians' professional networks and their attitudes towards EBM. *BMC Health Serv Res* 2013;*13*:429.

45. Mascia, D., Cicchetti, A., Fantini, M. P., et al. Physicians' propensity to collaborate and their attitude towards EBM: a cross-sectional study. *BMC Health Serv Res* 2011;*11*:172.

46. Mascia, D., Di Vincenzo, F., Iacopino, V., et al. Unfolding similarity in interphysician networks: the impact of institutional and professional homophily. *BMC Health Serv Res* 2015;*15*:8.

47. McCurdie, T., Sanderson, P., Aitken, L. M. Applying social network analysis to the examination of interruptions in healthcare. *Appl Ergon* 2018;*67*:50–60.

48. Meltzer, D., Chung, J., Khalili, P., et al. Exploring the use of social network methods in designing healthcare quality improvement teams. *Soc Sci Med* 2010;*71*(6):1119–1130.

49. Menchik, D. A., Meltzer, D. O. The Cultivation of Esteem and Retrieval of Scientific Knowledge in Physician Networks: Physician Networks. *J Health Soc Behav* 2010;*51*(2):137–152.

50. Myers, D. J., Lipscomb, H. J., Epling, C., et al. Surgical Team Stability and Risk of Sharps-Related Blood and Body Fluid Exposures During Surgical Procedures. *Infect Control Hosp Epidemiol* 2016;*37*(5):512–518.

51. Pappas, J M, Flaherty, K. E., Wooldridge, B. Achieving strategic consensus in the hospital setting: a middle management perspective. *Hospital Topics* 2003;*81*(1):15–22.

52. Pappas, J M, Wooldridge, B. Middle managers' divergent strategic activity: An investigation of multiple measures of network centrality. *J Manag Stud* 2007;*44*(3):323–341.

53. Pappas, J M, Flaherty, K. E., Wooldridge, B. Tapping into hospital champions--strategic middle managers. *Health Care Manag Rev* 2004;*29*(1):8–16.

54. Patterson, P. D., Pfeiffer, A. J., Weaver, M. D., et al. Network analysis of team communication in a busy emergency department. *BMC Health Serv Res* 2013;*13*:109.

55. Pinelli, V. A., Papp, K. K., Gonzalo, J. D. Interprofessional Communication Patterns During Patient Discharges: A Social Network Analysis. *J Gen Intern Med* 2015;*30*(9):1299–1306.

56. Rangachari, P. Knowledge sharing networks related to hospital quality measurement and reporting. *Health Care Manag Rev* 2008;*33*(3):253–263.

57. Reed Elliot, N. A blockmodel study of managerial hierarchies, verbal networks, and organizational culture in four hospitals. *Revista de Administração Pública* 2012;*46*(1):291–314.

58. Regts, G., Molleman, E. The moderating influence of personality on individual outcomes of social networks. *J Occup Organ Psychol* 2016;*89*(3):656–682.

59. Riley, R., Manias, E. Gatekeeping practices of nurses in operating rooms. *Soc Sci Med* 2009;*69*(2):215–222.

60. Shafiei, S. M., Azar, A., Esmaielpour, R. Mapping and social network analysis of the nurses of Razi hospital. *Iran Red Crescent Med J* 2018;*20(10):(n*(e58321).

61. Shoham, D. A., Harris, J. K., Mundt, M., et al. A network model of communication in an interprofessional team of healthcare professionals: A cross-sectional study of a burn unit. *J Interprof Care* 2016;*30*(5):661–667.

62. Shoham, D. A., Mundt, M. P., Gamelli, R. L., et al. The Social Network of a Burn Unit Team. *J Burn Care Res*, 2015;*36*(5):551–557.

63. Sreeramoju, P., Dura, L., Fernandez, M. E., et al. Using a Positive Deviance Approach to Influence the Culture of Patient Safety Related to Infection Prevention. *Open Forum Infect Dis* 2018;*5*(10):231.

64. Sykes, M., Gillespie, B. M., Chaboyer, W., et al. (2015). Surgical team mapping: implications for staff allocation and coordination. *AORN J* 2015;*101*(2):238–248.

65. Sykes, T. A., Venkatesh, V., Rai, A. Explaining physicians' use of EMR systems and performance in the shakedown phase. *JAMIA* 2011;*18*(2):125–130

66. Tasselli, S. Social networks and inter-professional knowledge transfer: The case of healthcare professionals. *Organ Stud* 2015;*36*(7):841–872.

67. Tsang, S. S., Chen, T. Y., Wang, S. F., et al. Nursing work stress: the impacts of social network structure and organizational citizenship behavior. *J Nurs Res* 2012;*20*(1):9–18.

68. Venkatesh, V., Zhang, X., Sykes, T. A. "Doctors do too little technology": A longitudinal field study of an electronic healthcare system implementation. *Inf Syst Res* 2011;*22*(3):523–546.

69. Wagter, J. M., van de Bunt, G., Honing, M., et al. Informal interprofessional learning: visualizing the clinical workplace. *J Interprof Care* 2012;*26*(3):173–182.

70. Walton, J. M., Steinert, Y. Patterns of interaction during rounds: implications for work-based learning. *Med Educ* 2010;*44*(6):550–558.

71. West, E., Barron, D. N. Social and geographical boundaries around senior nurse and physician leaders: an application of social network analysis. *Can J Nurs Res*, 2005;*37*(3):132–148.

72. West, E., Barron, D. N., Dowsett, J., et al. Hierarchies and cliques in the social networks of health care professionals: implications for the design of dissemination strategies. *Soc Sci Med* 1999;*48*(5):633–646.

73. Westbrook, J. I., Braithwaite, J., Georgiou, A., et al. Multimethod evaluation of information and communication technologies in health in the context of wicked problems and sociotechnical theory. *JAMIA* 2007;*14*(6):746–755.

74. Yi, W., Ben, C., Xitong, G., et al. Understanding User Adaptation toward a New IT System in Organizations: A Social Network Perspective. *J Assoc Inf Syst* 2017;*18*(11), 787–813.

75. Yuce, Y. K., Zayim, N., Oguz, B., et al. Analysis of social networks among physicians employed at a medical school. *Stud Health Technol Inform* 2014;*205*:543–547.

G) Table 2 (expanded version). Context-mechanism-outcome configurations with illustrative quotes from the literature

Context-mechanism–outcome configuration (CMOC)		Illustrative quotes
**DOMAIN 1: Social Groups** Close social ties formed with others, based on identity and personal view of the world (representation), in relation to the individual.		
CMOC1	When health care workers share a common identity (C), they will be more likely to trust one another (O), as they share an underlying representation (M).	*"Social tribes, like professional tribes, are split along professional lines. People are closer to and interact more readily with those with whom they identify, trained with, or share practices with, and to whom they are ideologically and attitudinally closer"* [1]
CMOC2a	When health care workers share a representation (C), they will preferentially communicate with one another (M) leading to insular communication (O).	*"Our first result is that homophily matters in the formation of connections within health care organizations. ….. People expect, a priori, that self-similar colleagues are more likely to accept them, be trustworthy, and hold similar beliefs, thereby mitigating the potential conflicts, misunderstandings, and monitoring costs that come with making connections"* [2]
CMOC2b	When health care workers share a representation (C), outsiders are easily identified (M), leading to communication boundaries (O).	*"Categories of professionals are typically separated from each other by social and cognitive boundaries, which may be an impediment to the creation of trustworthy relationships."* [2]
CMOC3	When a health care worker has an existing reciprocal relationship with a peer (C), they will preferentially seek advice or support from that person (O), because they trust the person (M1) and feel comfortable (M2).	*"Consistent across hospitals were significant positive effects for reciprocal relationships, indicating that there are pairs of professionals that tend to consult each other (mutual)."* [3]
CMOC4	When health care workers share a representation (C), this can lead to redundant information (O1), group-think (O2) and echo (O3), due to insular communication (M).	*"Physicians who were highly constrained in their interpersonal networks were less likely to self-report adopting EBM. This finding is consistent with the theory of structural holes (Burt, 1992). …One important risk is that subjects may start to trust their group judgment more than information from the surrounding scientific world."* [4]
CMOC5a	When a health care worker is central within a social group (C), their personal behaviours will embody group norms (O) because they will hold tightly to group identity as own (M).	*"..there is a significant negative association between the physicians' propensity to use EBM [evidence-based medicine] and the coreness they exhibit in their organization…the core is formed by physicians having a significantly lower propensity towards EBM than their peers located in the peripheral part of the network."* [5]

Context-mechanism–outcome configuration (CMOC)		Illustrative quotes
CMOC5b	When a health care worker is peripheral within a social group (C), they may deviate in behaviour from group norms (O1), and oppose/conflict with central members of the group (O2) because they may not wholly identify with the group representation (M).	*"… less-central individuals are likely exposed to fewer negative comments about the system. Thus, less-central individuals are more likely to adopt and use the new system."*[6]
CMOC6a	When a health care worker is central within a social group (C), they will have greater power over group norms (O), because they have greater influence over group narratives and representation (M).	*"Findings suggest that the more centrally located a manager is in a social cluster, the more likely that manager is to integrate new ideas into new organizational capabilities."*[7]
CMOC6b	When a health care worker is peripheral within a social group (C), they will have little power over group norms (O), because they have little influence over group narratives and representation (M).	*"…structure of this model underlies a group tightly connected physicians who interact strongly in order to exchange relevant knowledge, and a large number of less cohesive clinicians who are more likely to be connected amongst themselves than to members of the core part of the network. This result might be interpreted as a marginalization of physicians who are more prone to use EBM in their clinical practice."*[5]
CMOC6c	When a health care worker is central within a social group (C1) and has power over material resources (C2), they will have additional power over group norms (O) because they own the distribution of material resources and decision-making authority (M).	*"Nurse coordinators in operating rooms often exercised a dominating, hierarchical form of power in relation to surgeons as they approved or denied access to space, time and resources for surgical operations, based on the urgency of individual cases"*[8]<br><br>*"In the present case, high centrality occurred with department heads, such as the high connection density in Figure 2. In general, those with high work network centrality were those experienced nurses who not only controlled physical resources but were also in command of decision-making powers."*[9]
CMOC7a	Where a health care worker requires diverse information to undertake their role effectively, e.g. senior nurse, junior doctor (C), they will become more central in the workplace information-sharing network (O), because they must frequently seek information from others (M1) and others frequently seek information from them (M).	*"Centrality was not linked to gender, age and seniority in psychiatry, but was associated with job and activity."*[10]
CMOC7b	When a health care worker is peripheral in the workplace information-sharing network (C), they risk isolation (O), because they are not actively engaged in information-seeking (M1) or information-sharing (M2) with colleagues.	*"The communication network for Unit 5 has seven isolates. In addition, two of the more influential RNs (high Eigenvector Centrality) are not communicating with staff not on their shift, and there are a number of "pendants" (people with single links). The pendants are usually PCTs"*[11]

**Context-mechanism–outcome configuration (CMOC)**		**Illustrative quotes**
CMOC7c	When a health care worker needs legitimate/defensible information (C), they will speak to someone who embodies desirable group norms (O), because the information received will comply with social expectations (M).	*“For example, core RNs [registered nurses] can serve an important function in the communication of norms and process variations to noncore members such as anesthesia residents or other nursing staff members”*[12]
**<u>DOMAIN 2: Hierarchy</u>**<br>**Social ties or barriers formed based on hierarchical status of the individual.**		
CMOC8a:	When an individual is senior in the hierarchy (C1), or is a custodian of hierarchical status (C2), they have permission to approach both high and low status colleagues (O1) because of understanding of the ‘social rules of operation’ (M).	*“In particular, junior doctors collaborate with senior nurses who may then distribute information through clear lines of authority within the nursing profession.”*[3]<br><br>*“Thus it is by virtue of the hierarchical position in nurses’ networks provided by their organizational rank that nurse managers are allowed to actively control knowledge transfer between other nurses and the doctors.”*[13]<br><br>*“Junior doctors tend to borrow social capital from senior doctors”*[13]
CMOC8b:	When an individual is lower in the chain-of-command/hierarchy (C), they lack permission to approach some colleagues directly so accesses new/external information through the established chain of command (O1), or through a broker (O2), because of understanding of the ‘social rules of operation’ (M).	*“…in poor-coding facilities, the quality administrator functioned as a department manager, lacking formal authority at the organizational level. She said, ‘‘my role on the core measures is to provide data to the chief of medicine. I do not have a say in instituting changes related to practice and documentation”.”*[14]
CMOC9a	Where hierarchical control is paramount to the functioning of a social group (C) members will be more likely to accept and follow orders from a senior (O) due to concentration of authority (M). - a	*“In a centralised system, communication tends to flow vertically, between layers of the organisational hierarchy. That nurses' networks are more centralised tends to support the commonly held belief that hierarchical relations are more typical of this profession than medicine where the autonomy and independence of individual practitioners is emphasised.”*[15]
CMOC9b	Where hierarchical control is not paramount to the functioning of a social group (C) members will need to be convinced to change behaviour (O) due to flattened structure and distributed authority (M). - b	*“Cascading information from the top down may work for the nursing profession, especially if your first point of contact is a director of nursing. They have access to information and their networks are far-reaching. Certain behaviours which are acceptable in a hierarchy, such as orders, would not be acceptable in the more egalitarian structure of medical communities.”*[15]

Context-mechanism–outcome configuration (CMOC)		Illustrative quotes
CMOC10a	Where parallel hierarchies are operating within the same workplace (C), permission to approach colleagues outside one's own hierarchy structure will vary (O), according to the specific authoritative power over the individual and their understanding of the 'social rules of operation' between hierarchies (M).	*"Interprofessional team members recognized in the register nurse a leadership that facilitated their relationship with the physician. One physical therapist expressed "they know everything that happens, they do not have problem in speaking strong, in approaching a physician. Many other people panic about talking to a physician"."*[16]
CMOC10b	Where doctors have historically taken the role of leader in health care teams (C), they will assume a position of authority (O), because of their understanding of the 'social rules of operation' (M).	*"In the NICU, physicians serve as team leaders with ultimate professional and legal responsibility for patients."*[17]<br><br>*"... with doctors at the top of the clinical hierarchy, others (e.g., paraprofessionals) act in ways to preserve the hierarchy and defer to doctors' judgments."*[6]
CMOC11a	When there is a hierarchical structure (C), there is a risk of fragmentation into sub-groups based on status (O), because individuals identify more closely with peers of the same status (M1) and trust them more (M2).	*"However, many of the junior doctors are located in close proximity to each other, peripherally, on the left of the network. Less experienced medical staff seem thus to rely on each other rather than ask more senior staff for help to solve work-related problems."*[1]
CMOC11b	When an individual is higher up in the chain-of-command/hierarchy (C), they will preferentially communicate with other higher status staff (O), because they feel higher status colleagues will better understand their scope of work (M1), and will hold more useful information (M2).	*"In conclusion, there is evidence of teamwork occuring within multidisciplinary long-term care teams, but the type of teamwork differs for different staff groups. The collaborative teamwork described in the literature is limited to the higher status professionals."*[18]
CMOC11c	When an individual is lower in the chain-of-command/hierarchy (C), they will only expect specific types of support from colleagues of higher status (O), because of their understanding of the 'social rules of operation' (M1) and because they feel more comfortable with peers for usual support (M2).	*"Because they have clear definitions of what functions the different levels of managers should perform, communication between staff and managers can be enhanced by periodically surveying (formally or informally) staff about functional supports. Open, regular communication between staff and management will enhance satisfaction with manager performance."*[19]
CMOC12a	When an individual is higher up in the chain-of-command/hierarchy (C), they will preferentially communicate with other higher status staff (O), because they will further enhance their own status/power by association (M).	*"The structures reinforce the status differentials inherent in the teams and perpetuate the control of decision-making and problem-solving by the higher status professionals by ensuring that decision-making remains the onus of the higher status professionals. This is echoed in the formal organization of the teams in that only the higher status nurses and core multidisciplinary professionals attend team rounds on a regular basis."*[18]

Context-mechanism–outcome configuration (CMOC)		Illustrative quotes
CMOC12b	When an individual is higher up in the chain-of-command/hierarchy (C), they will have power over information (O) by controlling the information and resources disseminated to low status colleagues (M).	*"Gatekeeping was used by nurses to negotiate their social positioning in the health care hierarchy, both in relation to one another and with their medical colleagues. Nurse managers exercised control of information to reinforce their position in the nursing hierarchy (nurse managers have administrative responsibility for an operating room department and do not usually assume a clinical role)."*[8]
**<u>DOMAIN 3: Bridging distance</u>**<br>**The ways in which an individual can be encouraged to communicate with others with whom they do not have a close social tie.**		
CMOC13	When a health care worker observes a non-peer colleague displaying they are being 'careful' (C), they will exchange required information with this colleague (O), because they feel trusting towards the colleague (M1) and feel comfortable approaching them (M2).	*"Relational influencers are typically influential because of their 'people skills' and relational and communication expertise."*[20]
CMOC14	When a health care worker has a longstanding or multiplex relationship with a non-peer colleague (C), they will approach that colleague as a 'go-to person' (O), because of mutual understanding (M1), and trust (M2).	*"…the dietitian and social worker are long-term members of the team, having worked with the unit for many years. While it makes sense that a dietitian and a social worker are critical team members, we can only uncover their centrality in discussions about patient care using SNA."*[21]
CMOC15	When a health worker needs information urgently (C), they will select a colleague from those who are physically present (O), because they will receive an immediate response (M).	*"Collaboration and sharing knowledge and information are strongly influenced by physical context: the farther people are from each other, the less likely this interaction becomes (Cross & Parker, 2004). In this study, the two separate parts of the ward are clearly seen in the network structure: MCU and ICU."*[22]
CMOC16	When a health care worker can speak to a colleague face-to-face (C), information exchange will be enhanced (O), because they can better read and respond to verbal and non-verbal cues (M1) and is more confident that they will be understood (M2).	*"Supporting earlier findings, verbal communication was preferred by most participants and supports the importance of "common ground" between users for communication to be effective [82,85]. In person verbal communication includes the use of a common language and communication patterns such as body language that enhance the communication process."*[23]
CMOC17	Information can be exchanged between disconnected groups (O), when there is an individual who can act as a broker (C), because they communicate with members of both groups (M).	*"All of the blocks report getting and giving most of their information from either the higher status nurses (Block 1) or from other members of their own block. As such, the higher status nurses are the conduit through which information is passed between shifts and between the core multidisciplinary professionals (Block 5) and the nursing staff (Blocks 2-4). These higher status nurses are, therefore, the key linking or bridging persons between the blocks."*[18]<br><br>*"Directors of nursing then are the go-between for a number of people who would not otherwise be connected. There are good reasons for this in terms of their structural position in the formal organisation. As the average of their centrality scores indicate, nurses in this sample are in a*

Context-mechanism–outcome configuration (CMOC)		Illustrative quotes
		position to mediate and control, not just passively receive, information. The nurses in our sample occupy central positions which means that they play a very important role in the diffusion of information and influence within the wider structure of their profession by sending, receiving and transferring signals from diverse groups."[15]
**DOMAIN 4: Discourse** **The totality of the different ways of seeing the world, both content but also how each relates to the other and is communicated, in terms of overall dominance and power.**		
CMOC18a	The pattern of ongoing exchange and negotiation between social groups in the hospital workforce (C) creates and maintains (O1) or challenges (O2) the dominant discourse in a hospital, by demonstrating and exerting the relative power of different group representations within the hospital workforce (M).	*"The data in Table 2 suggest a "center-periphery" pattern similar to the pattern of interactions found among physicists (Cole & Cole, 1973), mathematicians (Crane, 1972), and biomedical researchers (Breiger, 1976). These studies indicate that groups of scientists and professionals are generally not organized in a hierarchical structure in which each person is directly accountable to a superior. Instead a group of influential individuals influences the direction of others' work and controls the distribution of resources while other professionals exchange information and resources with this central group directly or indirectly (Blissett, 1972). These results suggest such a pattern."*[24]
CMOC18b	For health care workers in an organisation, its rituals and norms (C) construct what an individual perceives is acceptable in the workplace (O), through the demonstration of social expectations and etiquettes (M).	*"… the network analysis of communication revealed that the average team briefings exhibited a hierarchical structure to communication, with the surgeon speaking the most frequently and dominating the conversation. On average, team members only communicated directly with the surgeon or resident and did not speak to each other during the briefing."*[25]
CMOC19a	When a health care worker has to do or say something at work (C), they keep to within a 'bandwidth' of actions they perceive to be 'accepted' or 'permitted' (O), based on their understanding of what the dominant discourse is within a hospital and their relative personal position based on identity and hierarchical status (M).	*""…The dialysis unit became the poster child for hand hygiene." A sign on the door of an isolation room at one end of the dialysis unit is another indication of change. When 4 doctors tried to enter the isolation room without proper garb, a nurse blocked the door and refused to budge until they donned gowns and gloves. As they re-entered the unit, she reminded them to dispose of their gowns and gloves in the bin in the isolation room. Then she gave them hand sanitizer. The sign, suggested by staff, reads: "STOP. Do Not Enter. Please See Staff." Staff prepared an infection control protocol to give to anyone entering the room."*[26]
CMOC19b	The perceived values of an organisation (C) facilitate the creation of status differentials within the workplace (O) because individuals benchmark their activities and identities against how much they embody these values (M).	*"… esteem is tied to work tasks. At top hospitals the focus is on removing doctors from the clinic so they can capitalize on their pedigree by publishing and speaking at conferences and grand rounds….. In the lower prestige hospitals the esteemed physicians sustain the local patient base"*[27]

Context-mechanism–outcome configuration (CMOC)		Illustrative quotes
CMOC20a	Where formal communication processes in hospitals are dysfunctional/unreliable (C), make-shift processes of informal information exchange dependent on social connections result (O) because individuals have to compensate to acquire necessary information (M).	*"Consistent with earlier findings, the providers in this study used non-linear strategies, such as incorporating multiple methods of communication to multiple providers simultaneously, to transfer or assume care of the patient [79]. This process has been identified as "channel switching" – using multiple sources of information to supplement one another in an attempt to piece together and understand the complex history and the current and future needs of the patient [80]."*[23]<br><br>*"..we have identified a gap in the structures of medicine and nursing, where there are few informal ties. This means that information is unlikely to be spread between the two professions by informal routes. This makes formal arrangements for communication all the more important."*[28]
CMOC20b	When informal information exchange becomes the norm in a hospital (C), access to information by specific individuals and groups is inherently skewed (O), because it will depend on how socially connected the individual or group is (M).	*"Providers working in inpatient units reported that they were unhappy with their ability to communicate with providers on different shifts. For example, a nurse working the night shift in an inpatient unit stated, "One of my biggest frustrations is the inability to discuss with the primary physician team my concerns/observations/patient requests that do not warrant a call to the covering team." This provider stated that in this situation she either had to ask the day nurse to convey her concerns or tape a note to the front of the chart with the hope the physician would read it"*[23]

References:

1. Creswick N, Westbrook JI, Braithwaite J. Understanding communication networks in the emergency department. BMC Health Serv Res 2009;9:247.
2. Mascia D, Di Vincenzo F, Iacopino V, et al. Unfolding similarity in interphysician networks: the impact of institutional and professional homophily. BMC Health Serv Res. 2015;15:8.
3. Chan B, Reeve E, Matthews S, et al. Medicine information exchange networks among health care professionals and prescribing in geriatric medicine wards. Br J Clin Pharmacol. 2017;83(6):1185–96.
4. Mascia D, Cicchetti A. Physician social capital and the reported adoption of evidence-based medicine: exploring the role of structural holes. Soc Sci Med. 2011 Mar;72(5):798–805.
5. Mascia D, Cicchetti A, Damiani G. "Us and them": a social network analysis of physicians' professional networks and their attitudes towards EBM. BMC Health Serv Res 2013;13:429.
6. Venkatesh V, Zhang X, Sykes TA. "Doctors do too little technology": A longitudinal field study of an electronic health care system implementation. Inf Syst Res. 2011;22(3):523–46.

7. Pappas JM, Flaherty KE, Wooldridge B. Tapping into hospital champions--strategic middle managers. Health Care Manage Rev. 2004;29(1):8–16.

8. Riley R, Manias E. Gatekeeping practices of nurses in operating rooms. Soc Sci Med 2009;69(2):215–22. 9.

9. Tsang SS, Chen TY, Wang SF, et al. Nursing work stress: the impacts of social network structure and organizational citizenship behavior. J Nurs Res 2012;20(1):9–18.

10. Boyer L, Belzeaux R, Maurel O, et al. A social network analysis of health care professional relationships in a French hospital. Int J Health Care Qual Assur 2010;23(5):460–9. 11.

11. Effken JA, Gephart SM, Brewer BB, et al. Using *ORA, a network analysis tool, to assess the relationship of handoffs to quality and safety outcomes. CIN Comput Informatics, Nurs 2013;31(1):36–44. 12.

12. Anderson C, Talsma A. Characterizing the structure of operating room staffing using Social Network Analysis. Nurs Res. 2011;60(6):378–85.

13. Tasselli S. Social networks and inter-professional knowledge transfer: The case of health care professionals. Organ Stud 2015;36(7):841–72. 14.

14. Rangachari P. Knowledge sharing networks related to hospital quality measurement and reporting. Health Care Manage Rev 2008;33(3):253–63.

15. West E, Barron DN, Dowsett J, et al. Hierarchies and cliques in the social networks of health care professionals: implications for the design of dissemination strategies. Soc Sci Med 1999;48(5):633–46.

16. Espinoza P, Peduzzi M, Agreli H, et al. Interprofessional team member's satisfaction: a mixed methods study of a Chilean hospital. Hum Resour Health. 2018;16:30.

17. Grace MK, VanHeuvelen JS. Ties Received, Support Perceived: A Test of the Theorized Relationships among Workplace Networks, Social Support, and Mental Health in a Neonatal Intensive Care Unit (NICU). Soc Ment Health. 2015;5(2):106–27.

18. Cott C. "We decide, you carry it out": a social network analysis of multidisciplinary long-term care teams. Soc Sci Med 1997;45(9):1411–21.

19. MacPhee M, Scott J. The role of social support networks for rural hospital nurses: supporting and sustaining the rural nursing work force. J Nurs Adm. 2002 May;32(5):264–72.

20. Fong A, Clark L, Cheng T, et al. Identifying influential individuals on intensive care units: using cluster analysis to explore culture. J Nurs Manag. 2017;25(5):384–91.

21. Shoham DA, Mundt MP, Gamelli RL, et al. The Social Network of a Burn Unit Team. J Burn Care Res 2015;36(5):551–7.

22. Wagter JM, van de Bunt G, Honing M, et al. Informal interprofessional learning: visualizing the clinical workplace. J Interprof Care 2012;26(3):173–82.

23. Benham-Hutchins MM, Effken JA. Multi-professional patterns and methods of communication during patient handoffs. Int J Med Inform 2010;79(4):252–67.

24. Anderson JG, Jay SJ. Physician Utilization of Computers: A Network Analysis of the Diffusion Process. J Organ Behav Manage 1985;6(3,4):21. 25.

25. Forsyth KL, Hildebrand EA, Hallbeck MS, et al. Characteristics of team briefings in gynecological surgery. Appl Ergon. 2019;78:263-269

26. Lindberg C, Downham G, Buscell P, et al. Embracing collaboration: a novel strategy for reducing bloodstream infections in outpatient hemodialysis centers. Am J Infect Control 2013;41(6):513–9. 27.

27. Menchik DA, Meltzer DO. The Cultivation of Esteem and Retrieval of Scientific Knowledge in Physician Networks: Physician Networks. J Health Soc Behav 2010;51(2):137–52.

28. West E, Barron DN. Social and geographical boundaries around senior nurse and physician leaders: an application of social network analysis. Can J Nurs Res 2005;37(3):132–48.
